# Resection rates after conversion therapy for initially unresectable hepatocellular carcinoma: a systematic review and meta-analysis

**DOI:** 10.3389/fonc.2026.1898480

**Published:** 2026-10-01

**Authors:** Xiaobo Ouyang, Ji Li, Po Liang, Yanfei Wang, Yangming Liu, Zhuoren Cheng, Ping Che

**Affiliations:** 1 Department of Gastroenterology, People’s Hospital of Chongqing Hechuan, Chongqing, China; 2 Department of Hepatobiliary Surgery, People’s Hospital of Chongqing Hechuan, Chongqing, China; 3 Department of General Practice, People’s Hospital of Chongqing Hechuan, Chongqing, China; 4 Department of Obstetrics, The Affiliated Banan Hospital of Chongqing Medical University (People’s Hospital of Banan District), Chongqing, China; 5 Department of Hepatobiliary Surgery, Shapingba Hospital Affiliated to Chongqing University (Shapingba District People’s Hospital of Chongqing), Chongqing, China

**Keywords:** conversion therapy, hepatectomy, hepatocellular carcinoma, initially unresectable HCC, liver resection, meta-analysis, systematic review

## Abstract

**Introduction:**

Conversion therapy may create an opportunity for liver resection in selected patients with initially unresectable hepatocellular carcinoma (HCC), but reported conversion rates use heterogeneous denominators and endpoints. We estimated actual completed hepatectomy using a verified all-treated denominator.

**Methods:**

PubMed/MEDLINE, Embase, Web of Science Core Collection, Cochrane Library/CENTRAL, Wanfang Data, and CNKI were searched through April 10, 2026. Eligible cohorts included initially or baseline unresectable HCC, an all-treated denominator, and completed liver resection events. Two authors independently assessed eligibility, extracted data, and evaluated risk of bias. Random-effects logit proportion meta-analysis, Hartung-Knapp sensitivity, prediction intervals, leave-one-out analyses, treatment-strategy subgroups, publication-year meta-regression, and small-study-effect diagnostics were undertaken. PROSPERO: CRD420261360108.

**Results:**

Thirty-seven reports represented 36 unique studies; 34 studies involving 5,137 patients and 1,042 completed hepatectomies entered the quantitative synthesis. The crude aggregate resection rate was 20.3% (1,042/5,137), whereas the random-effects modeled pooled rate was 25.40% (95% CI, 20.74%-30.69%; I^2^=91.5%; tau^2^=0.5132). The Hartung-Knapp 95% CI was 20.12%-31.51%, and the prediction interval was 7.16%-60.04%. Leave-one-out estimates ranged from 24.52% to 26.64%. Treatment-strategy differences were not statistically significant (Qm=5.17, df=2, P = 0.075). Publication-year meta-regression did not show a clear linear association (OR per decade 1.19, 95% CI 0.77-1.83; P = 0.440), and Egger’s test did not indicate significant funnel asymmetry (P = 0.381).

**Conclusions:**

Approximately one-quarter of patients initiating conversion therapy underwent actual hepatectomy, but the crude and modeled estimates differ because substantial heterogeneity changes random-effects study weighting. The pooled estimate is a cross-study, cross-era benchmark for multidisciplinary decision-making rather than a universal patient-level probability or a treatment-specific efficacy estimate.

**Systematic Review Registration:**

www.crd.york.ac.uk/prospero/, identifier CRD420261360108.

## Introduction

Hepatocellular carcinoma (HCC) remains a major global health burden and a leading cause of cancer-related mortality. Liver resection can provide durable disease control in selected patients, but many patients present with tumors that are not suitable for curative-intent surgery because of tumor extent, vascular invasion, inadequate future liver remnant, impaired liver reserve, or unfavorable oncological features (1, 2).

The treatment landscape of unresectable HCC has changed with immune checkpoint inhibitors, antiangiogenic agents, molecular targeted therapies, and locoregional treatments such as transarterial chemoembolization (TACE), hepatic arterial infusion chemotherapy (HAIC), portal vein embolization-based strategies, and selective internal radiation therapy (3, 4).

Several recent meta-analyses have synthesized conversion-to-surgery outcomes in unresectable HCC. Pei et al. reported a pooled conversion rate of 25% for combined locoregional-systemic therapy; subsequent regimen-focused reviews reported rates of approximately 23%-35% for contemporary immune-targeted combinations (5–8). These estimates establish the clinical relevance of conversion therapy but are not directly interchangeable because the prior reviews differed in regimen restrictions, definitions of successful conversion, and whether radiological resectability, surgery eligibility, or completed resection constituted the event. Representative studies have reported actual liver resection after locoregional, systemic, and combined locoregional-systemic conversion strategies (9–16).

A denominator problem also remains. Studies restricted to surgery-treated patients or post-conversion hepatectomy cohorts provide important postoperative information but cannot estimate the probability of resection among all patients who initiate conversion therapy. Similarly, counting radiological downstaging, eligibility for surgery, transplantation, or ablation as conversion success yields a different estimand from completed liver resection.

This review therefore estimated the pooled actual liver resection rate after conversion or downstaging therapy using a verified all-treated denominator and an endpoint restricted to completed liver resection. Its incremental contribution is estimand standardization rather than an expectation of a numerically divergent pooled rate: the review applies a common denominator and event definition across regimen classes, includes English- and Chinese-language evidence through April 2026, and reports prediction intervals, influence analyses, small-study effects, and GRADE certainty.

## Methods

### Study design and registration

This systematic review was conducted and reported in accordance with PRISMA 2020 and relevant Cochrane guidance (17, 18). The initial review protocol was registered in PROSPERO on April 5, 2026 (CRD420261360108). At that time, searching/study identification had commenced but remained incomplete; formal eligibility screening, data extraction, risk-of-bias assessment, and quantitative synthesis had not commenced. Database searches were completed on April 10, 2026, and preliminary screening, full-text assessment, data extraction, and risk-of-bias assessment were undertaken between April 10 and May 1, 2026. The completed PRISMA 2020 checklist is provided in the **Supplementary Material**.

The initially registered primary objective was to evaluate predictors of successful conversion therapy. During preliminary extraction, candidate predictors were reviewed descriptively but were found to use incompatible definitions, thresholds, reference groups, effect measures, adjustment sets, and statistical models. No predictor meta-analysis was performed because a clinically and statistically defensible quantitative synthesis of predictors was not possible.

The primary quantitative outcome was amended in PROSPERO Version 2.0 on May 1, 2026, to completed liver resection among all patients initiating an eligible conversion or downstaging regimen. Because this change occurred after preliminary data extraction had begun, it is treated as a substantive protocol amendment rather than as an outcome prospectively specified at initial registration. The final outcome-specific study-level dataset, endpoint adjudications, and quantitative synthesis were completed after the amendment. The potential for *post hoc* outcome specification and selective emphasis arising from this timing is explicitly addressed as a limitation.

### Search strategy and eligibility criteria

Database-specific search strategies, platforms, search dates, and pre-deduplication record counts are reported in **Supplementary Table S2**. The original platform-generated search-history files or exports were not available in the archived project files at final assembly. Accordingly, the strategies in **Supplementary Table S2** are presented as the database-specific strategies recorded in the review materials and are not described as independently platform-verified exact histories.

Eligible studies enrolled patients with initially unresectable or baseline unresectable HCC who received conversion or downstaging therapy and reported surgical outcomes. Studies entered the primary analysis only when the denominator represented patients who initiated an eligible regimen and the numerator represented completed liver resection, hepatectomy, salvage liver resection, or conversion surgery. Liver transplantation, ablation, radiological conversion, eligibility for resection, and conversion without surgery were not counted as events. Historical downstaging cohorts were eligible when they enrolled an all-treated unresectable population, applied subsequent surgical reassessment, and reported completed liver resections. Eligibility was determined by the underlying cohort and extractable endpoint rather than the stated primary purpose of the source article; accordingly, prediction-model studies were eligible when their source cohort met the same population, denominator, and event criteria. The PICOS framework was: population, patients with initially or baseline unresectable HCC; intervention, conversion or downstaging therapy; comparator, not required for inclusion because eligible single-arm cohorts were permitted; outcome, completed liver resection among the all-treated population; and study design, prospective or retrospective trials or cohorts with an extractable all-treated denominator and completed-resection numerator.

Studies limited to patients who had already undergone surgery, studies without an all-treated denominator, duplicate publications, nonseparable mixed populations, and reports with insufficient endpoint information were excluded from the primary synthesis. A conservative report-selection hierarchy was applied to manage probable cohort overlap. The hierarchy was based on center coverage, recruitment span, denominator clarity, and reporting completeness and was applied without reference to the magnitude or statistical significance of the reported resection rate. Confirmed companion reports were counted once, and potentially overlapping reports not selected for primary pooling were retained only for unique descriptive information when informative; no uncertain overlapping report was allowed to expand the primary denominator.

### Study selection and data extraction

Two reviewers (X.O. and J.L.) independently screened titles, abstracts, and full-text reports against the predefined eligibility criteria. The same two reviewers independently re-extracted all synthesis-critical data for all 36 eligible studies from their 37 linked reports using separate copies of a standardized extraction form and without access to each other’s entries until both forms were completed. Independently extracted items included the all-treated denominator, completed liver resection events, treatment-arm data, endpoint definitions, secondary-outcome denominators, and information needed to identify duplicate or potentially overlapping cohorts. Study characteristics not entering a synthesis were independently verified by the second reviewer. The two forms were then compared item by item. Discrepancies were resolved by re-examination of the full text and **Supplementary Materials** and by consensus; disagreements not resolved by consensus were adjudicated by P.C. The independent forms, discrepancy log, adjudication decisions, and reconciled dataset were retained by the review team.

All quantitative analyses used a study-level dataset. When a study reported both liver resection and liver transplantation, only liver resection was counted. Radiological downstaging, ablation, transplantation, or conversion-to-resectability without a separate resection count was not counted as an event. Potential cohort overlap was assessed from centers, enrollment periods, authorship, regimens, sample sizes, and cohort characteristics. Confirmed companion reports from the same cohort were linked and counted once using a canonical report for primary extraction. When independence could not be established for a smaller network report, that report was retained only for descriptive outcomes and excluded from primary pooling; these report-selection decisions were made without reference to the magnitude or statistical significance of the reported resection rate. Multiarm studies remained single studies in the overall analysis but contributed arm-level data to treatment-strategy subgroups when the arms belonged to different classes.

### Management of potentially overlapping cohorts

Potential overlap was evaluated from publication-level evidence rather than inferred solely from shared authorship or institutions. Definite companion publications were linked and counted once. When independence could not be established for a smaller network report, that report was retained only for descriptive synthesis and excluded from primary pooling to avoid possible double counting. Because these report-linkage decisions were finalized after protocol registration, they are reported transparently as *post hoc* data-integrity decisions, and residual overlap is acknowledged as a limitation.

### Risk of bias assessment

Two reviewers (X.O. and J.L.) independently reassessed the 34 studies contributing to the primary quantitative synthesis using one design-appropriate appraisal tool. Comparative nonrandomized cohorts were evaluated with the Newcastle-Ottawa Scale (NOS), single-arm and other noncomparative cohorts with the Joanna Briggs Institute (JBI) case-series checklist, and the randomized FT059 study with RoB 2. Each reviewer recorded the source location and justification for every domain judgment before comparison. Discrepancies were resolved by source re-examination and consensus; unresolved disagreements were adjudicated by P.C. Domain-level judgments, rather than a cross-tool numerical score, informed interpretation and certainty assessment.

The NOS assessment covered cohort selection, comparability, outcome ascertainment, follow-up duration, and follow-up completeness. The JBI assessment covered inclusion criteria, case identification, outcome measurement, consecutive and complete inclusion, participant and clinical reporting, follow-up or outcome reporting, and statistical analysis. Domain-level supporting reasons and final consensus judgments are presented in **Supplementary Table S3** and **Supplementary Figures S2A-C**. No cross-instrument quality-threshold sensitivity analysis was performed because the instruments are not commensurate.

### Certainty of evidence assessment

Certainty of evidence for the primary outcome was assessed using the GRADE framework as adapted for noncomparative meta-analyses of proportions (19, 20). Because this review estimated an outcome probability rather than a comparative effect, GRADE was applied to the certainty of the pooled actual liver resection-rate estimate.

Evidence from nonrandomized and observational studies was initially rated as low certainty and then assessed for risk of bias, inconsistency, indirectness, imprecision, and publication bias or small-study effects. No upgrading was applied. The GRADE assessment is provided in **Supplementary Table S4**.

### Outcomes and statistical analysis

The primary outcome was the actual liver resection rate after conversion or downstaging therapy. This was defined as the proportion of patients in the eligible all-treated denominator who subsequently underwent liver resection, hepatectomy, salvage liver resection, or conversion surgery.

Secondary outcomes included overall survival, progression-free survival, recurrence-free survival, disease-free survival, recurrence, objective radiological response, pathological complete response, major pathological response, and postoperative outcomes. These outcomes were summarized descriptively rather than pooled because definitions, follow-up schedules, response criteria, and denominators varied across studies.

Pooled estimates were generated using inverse-variance random-effects meta-analysis of logit-transformed proportions. DerSimonian-Laird estimation of tau^2^ was retained as the primary model for analytic continuity; two-sided normal-theory 95% confidence intervals were used for the primary estimate. Heterogeneity was quantified using I^2^ and tau^2^, and a t-based 95% prediction interval was calculated. A DerSimonian-Laird Hartung-Knapp confidence interval was calculated as an inferential sensitivity analysis.

Funnel-plot inspection and Egger’s regression were used to assess small-study effects (**Figure 1**). These diagnostics were interpreted cautiously because substantial clinical and statistical heterogeneity can limit the validity and power of funnel-plot asymmetry tests in meta-analyses of proportions.

**Figure 1:**
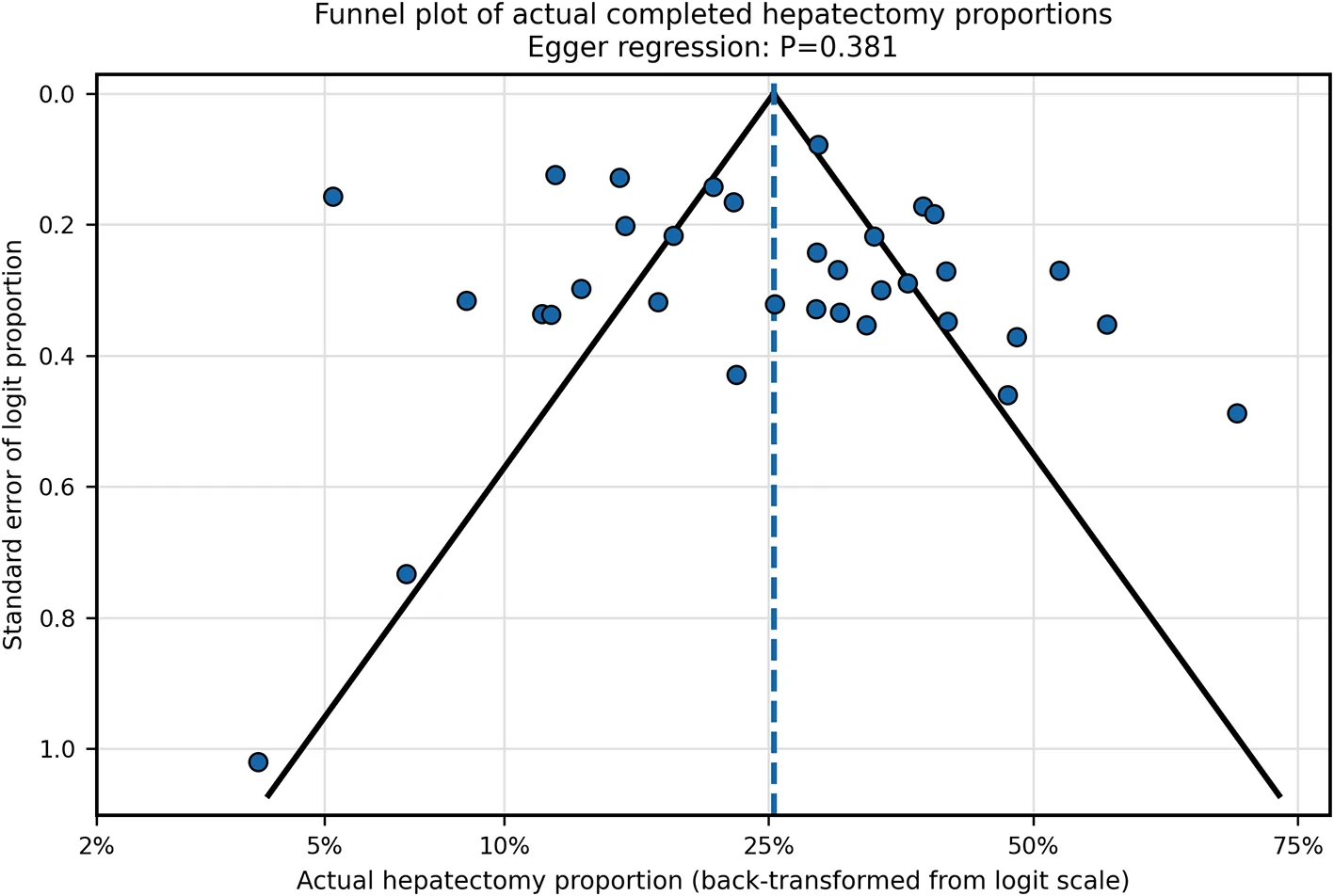
Funnel plot of logit-transformed actual hepatectomy proportions against standard error, with back-transformed proportion tick labels. Egger’s regression did not indicate significant asymmetry (P = 0.381).

An exploratory mixed-effects meta-regression used treatment strategy as a categorical study-arm moderator with a common DerSimonian-Laird tau^2^ estimate; the omnibus Qm test was used only to explore heterogeneity and not for causal inference. In addition to the random-effects estimate, the crude aggregate resection rate was calculated as the total number of completed hepatectomies divided by the total all-treated denominator across the 34 primary-analysis studies. To address the broad 1998–2026 publication span beyond the existing historical-study sensitivity analyses, a *post hoc* exploratory univariable random-effects meta-regression used publication year as a continuous study-level moderator. Publication year was treated only as an ecological proxy for treatment era. Primary study-level estimates and the publication-year analysis were independently re-verified using Python 3.12 with NumPy, pandas, and SciPy. The final 34-study analytic dataset, data dictionary, verification code, and supporting outputs are publicly available in Zenodo (DOI: 10.5281/zenodo.21976635).

Exploratory subgroup analyses used three mutually exclusive treatment classes: systemic therapy alone; locoregional therapy alone; and locoregional plus systemic therapy. Multiarm studies remained single studies in the overall analysis. For the strategy analysis, arms were separated only when they belonged to different classes; arms from the same parent study and class were combined before meta-analysis. Zhang X 2026, Lin SR 2025, Yang DL 2025 (GUIDANCE001), and Du Y 2023 contributed class-specific arm-level data. Piao M 2025 remained in the overall analysis but was excluded from the strategy analysis because the source cohort’s variable locoregional components could not be assigned consistently. The final strategy analysis comprised 37 independent class-specific units from 33 parent studies.

Sensitivity analyses evaluated a broader reported conversion-resection definition including FT020, contemporary modalities, the pre-existing highest source-verification category (Evidence Tier A), exclusion of the historical iodine-131 study FT058, exclusion of the historical high-dose TACE study FT059, and exclusion of the historical broad TACE-downstaging cohort FT011. Evidence-tier labels were source-verification categories from the review audit and were distinct from risk-of-bias judgments. Leave-one-out analyses assessed the influence of every study in the primary synthesis.

## Results

### Study selection

A total of 956 records were identified from PubMed/MEDLINE (n=149), Web of Science Core Collection (n=155), Cochrane Library/CENTRAL (n=167), Embase (n=267), Wanfang Data (n=215), and CNKI (n=3). After removal of 80 duplicates, 876 records were screened and 802 were excluded at title/abstract screening. Seventy-four database reports underwent full-text assessment; one additional canonical/companion report was identified through citation and source reconciliation.

Among the 74 database reports assessed for eligibility, 38 were excluded because they were nonoriginal or insufficient reports (n=10), had endpoint mismatch or no extractable actual resection outcome (n=11), represented duplicate or overlapping cohorts or data sources (n=7), did not evaluate a relevant conversion-therapy strategy or used a historical noncomparable modality (n=3), lacked an all-treated denominator or included only post-conversion surgery cohorts (n=3), had a wrong disease population or unconfirmed HCC denominator (n=2), or included an ineligible or nonseparable mixed population (n=2). The single additional report identified through citation and source reconciliation was assessed separately and retained.

Thirty-seven eligible reports represented 36 unique studies. One 94-patient study was reported in two linked publications (Li X 2023 and Li X 2024) (21, 22); these were treated as a single study and the more complete canonical report was used for primary extraction, while the companion report supplied secondary context. Thirty-four independent studies entered the primary quantitative synthesis. FT020 was retained qualitatively because its reported 3/26 conversion-resection endpoint did not explicitly document completed hepatectomy, and the smaller Wei et al., 2021 network report (12) was retained descriptively because independence from a larger multicenter network cohort could not be confirmed (**Figure 2**).

**Figure 2:**
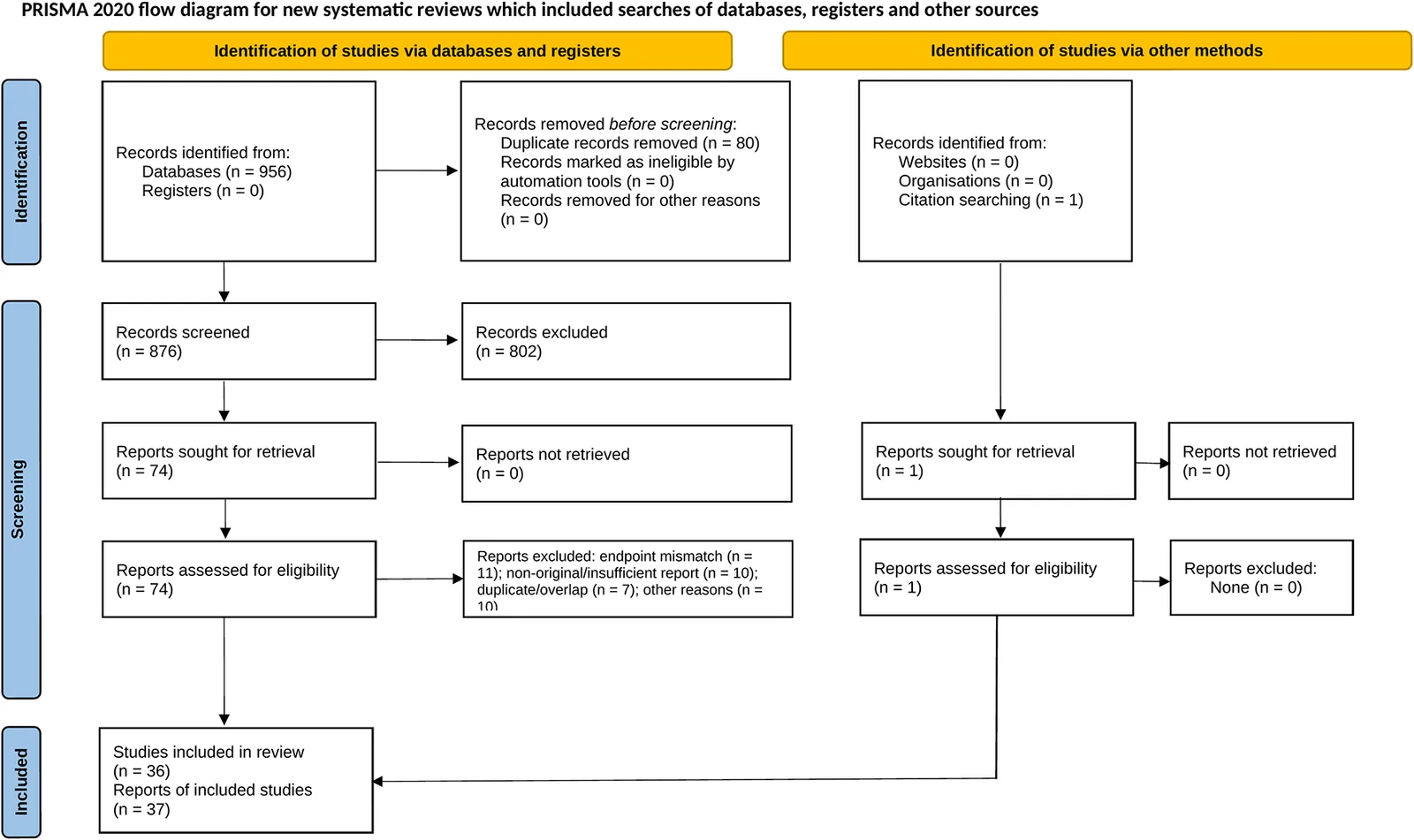
PRISMA 2020 flow diagram. A total of 956 database records were identified and 80 duplicates were removed. After screening 876 records, 74 database reports underwent full-text assessment; one additional canonical/companion report was identified through citation tracing. Thirty-seven reports represented 36 unique studies, of which 34 entered the quantitative synthesis. Adapted from the official PRISMA 2020 flow-diagram template (Page et al., BMJ 2021;372:n71; 17), licensed under CC BY 4.0.

### Study characteristics

The 34 studies contributing to the primary synthesis comprised 5,137 patients and 1,042 completed hepatectomies and were published between 1998 and 2026. Thirty-two studies were conducted in China, one in France, and one in the United States. Study-level endpoint data are shown in **Table 1**, with detailed evidence and source notes in **Supplementary Table S1**.

**Table 1:** Study-level data included in the primary quantitative synthesis.

Ref.	Study	Country/region	Study design	Dominant regimen class	N	Resection n	Rate
(23)	Lu YC 2024	China	Retrospective cohort	LR+SYS	51	13	25.5%
(24)	Bai Z 2024	China	Retrospective cohort	LR+SYS	31	7	22.6%
(25)	Zhang X 2026	China	Prospective multicenter nonrandomized phase II	LR/LR+SYS	142	55	38.7%
(26)	Pan X 2023	China	Retrospective cohort	LR+SYS	49	17	34.7%
(15)	He C 2022	China	Retrospective comparative cohort	LR	33	19	57.6%
(27)	Su Y 2026	China	Retrospective model-development cohort	LR+SYS	210	47	22.4%
(9)	Zhang YQ 2016	China	Retrospective cohort	LR	831	43	5.2%
(28)	Lin SR 2025	China	Retrospective multicenter cohort	LR/LR+SYS	301	63	20.9%
(29)	Wu XK 2024	China	Prospective multicenter single-arm	LR+SYS	55	29	52.7%
(30)	Zhang W 2023	China	Prospective single-arm phase II	SYS	56	23	41.1%
(31)	Wang L 2023	China	Prospective phase II	SYS	36	12	33.3%
(32)	Regnault H 2024	France	Retrospective cohort	LR	127	11	8.7%
(33)	Zhang YC 2023	China	Retrospective cohort	LR+SYS	42	13	31.0%
(34)	Yang DL 2025 GUIDANCE001	China	Multicenter retrospective cohort	LR/LR+SYS	802	233	29.1%
(35)	Du Y 2023	China	Retrospective comparative cohort	LR/LR+SYS	29	2	6.9%
(36)	Li J 2023	China	Retrospective cohort	LR+SYS	87	10	11.5%
(37)	He ZL 2026	China	Observational cohort; timing not reported	LR+SYS	45	13	28.9%
(38)	Piao M 2025	China	Retrospective cohort	Systemic-based; variable LR*	613	74	12.1%
(11)	Li B 2021	China	Retrospective comparative cohort	LR	83	24	28.9%
(13)	Zhang J 2021	China	Retrospective cohort	LR+SYS	34	14	41.2%
(39)	Yang YG 2023	China	Retrospective comparative cohort	LR+SYS	69	12	17.4%
(40)	Zeng ST 2025	China	Retrospective comparative cohort	LR+SYS	84	10	11.9%
(41)	Yalikun K 2025	China	Prospective single-arm exploratory trial	LR+SYS	19	9	47.4%
(16)	Qu WF 2022	China	Retrospective real-world cohort	LR+SYS	51	19	37.3%
(42)	Son SY 2024	United States	Retrospective cohort	LR	26	1	3.8%
(10)	Chen S 2022	China	Retrospective comparative cohort	LR+SYS	142	26	18.3%
(43)	Zeng ZC 1998	China	Prospective phase I/II trial	LR	65	20	30.8%
(44)	Chen MS 2002	China	Randomized trial	LR	473	72	15.2%
(45)	Liu D 2023	China	Prospective single-arm phase II	LR+SYS	29	14	48.3%
(46)	Gan LJ 2023	China	Retrospective cohort	LR+SYS	98	13	13.3%
(47)	Cao YJ 2023	China	Prospective single-arm phase II	LR+SYS	20	14	70.0%
(48)	Xu B 2022	China	Retrospective cohort	SYS	187	29	15.5%
(49)	Zhou Y 2025	China	Retrospective comparative cohort	LR+SYS (with/without RT)	123	49	39.8%
(22)	Li X 2024	China	Retrospective real-world cohort	LR+SYS	94	32	34.0%

Rates use the all-treated denominator and count only completed liver resection. Study designs report prospective/retrospective timing where documented. Detailed regimens, source identifiers, evidence tiers, and endpoint adjudications are provided in **Supplementary Table S1**.

LR, locoregional therapy alone; LR+SYS, locoregional plus systemic therapy; SYS, systemic therapy alone; RT, radiotherapy. *Piao M 2025 was retained in the overall analysis but excluded from strategy subgroups because variable locoregional components could not be assigned consistently.

Treatment approaches included systemic therapy, locoregional therapy, and combined regimens incorporating TACE, HAIC, portal vein embolization-based approaches, selective internal radiation therapy, tyrosine kinase inhibitors, anti-PD-1/PD-L1 antibodies, antiangiogenic agents, radiotherapy, and individualized multimodal combinations (9–16, 21, 23–34, 36–41, 45–47, 49–54). Study-level characteristics, including reference number, country/region, study design, and dominant regimen class, are summarized in **Table 1**; **Supplementary Table S1**.

Study-level endpoint data for the primary meta-analysis are shown in **Table 1**. Detailed study design, data source, regimen classification, sample size, event count, and extraction notes are provided in **Supplementary Table S1**.

### Risk of bias and certainty of evidence

Among the 14 comparative nonrandomized studies assessed with the NOS, scores ranged from 4 to 8 and the most frequent concerns involved comparability and follow-up. Among the 19 noncomparative cohorts assessed with the JBI checklist, one met all 10 criteria, seven met 8-9, six met 7, and five met 3–6 criteria; limitations most often involved consecutive or complete inclusion and outcome/follow-up reporting. The randomized FT059 study was judged at high overall risk under RoB 2 because the randomization process was insufficiently described.

No eligible report was excluded solely because of risk of bias. Domain-level judgments were used to contextualize the pooled estimate and GRADE certainty rather than to create a cross-tool low-risk subset (**Supplementary Table S3** and **Supplementary Figures S2A–C**).

The certainty of evidence for the pooled actual hepatectomy rate was judged very low. The main reasons were serious risk of bias, very serious inconsistency (I^2^=91.5% and a wide prediction interval), and serious indirectness from variable definitions of unresectability, conversion intent, and surgical candidacy. Egger’s test did not indicate statistically significant funnel asymmetry (P = 0.381); publication bias was therefore not rated down separately. The pooled rate should be interpreted as a study-level benchmark rather than a universal patient-level probability (**Supplementary Table S4**).

### Primary outcome: pooled actual liver resection rate

The primary pooled analysis included 34 studies contributing 5,137 patients and 1,042 completed hepatectomies. The crude aggregate resection rate was 20.3% (1,042/5,137). Source-level verification corrected four endpoint records before this synthesis: Du Y 2023 (2/29; one ablation excluded), Li B 2021 (24/83), Gan LJ 2023 (13/98; two RFA procedures excluded), and Cao YJ 2023 (14/20).

The DerSimonian-Laird random-effects modeled actual hepatectomy rate was 25.40% (95% CI, 20.74%-30.69%). This estimate is not a recalculation of the crude 20.3% aggregate proportion: under substantial between-study heterogeneity, random-effects weights incorporate within-study uncertainty and between-study variance rather than weighting studies solely by their participant counts. The DerSimonian-Laird Hartung-Knapp 95% CI was 20.12%-31.51% (**Table 2**; **Figure 3**).

**Table 2:** Summary of the primary random-effects meta-analysis.

Outcome	Studies	Participants/events	Pooled estimate	Heterogeneity	Additional statistics
Actual completed hepatectomy rate	34	5,137/1,042	25.40% (95% CI 20.74%-30.69%)	I^2^=91.5%; tau^2^=0.5132; PI 7.16%-60.04%	Crude aggregate rate 20.3% (1,042/5,137); HK 95% CI 20.12%-31.51%; Egger P = 0.381

**Figure 3:**
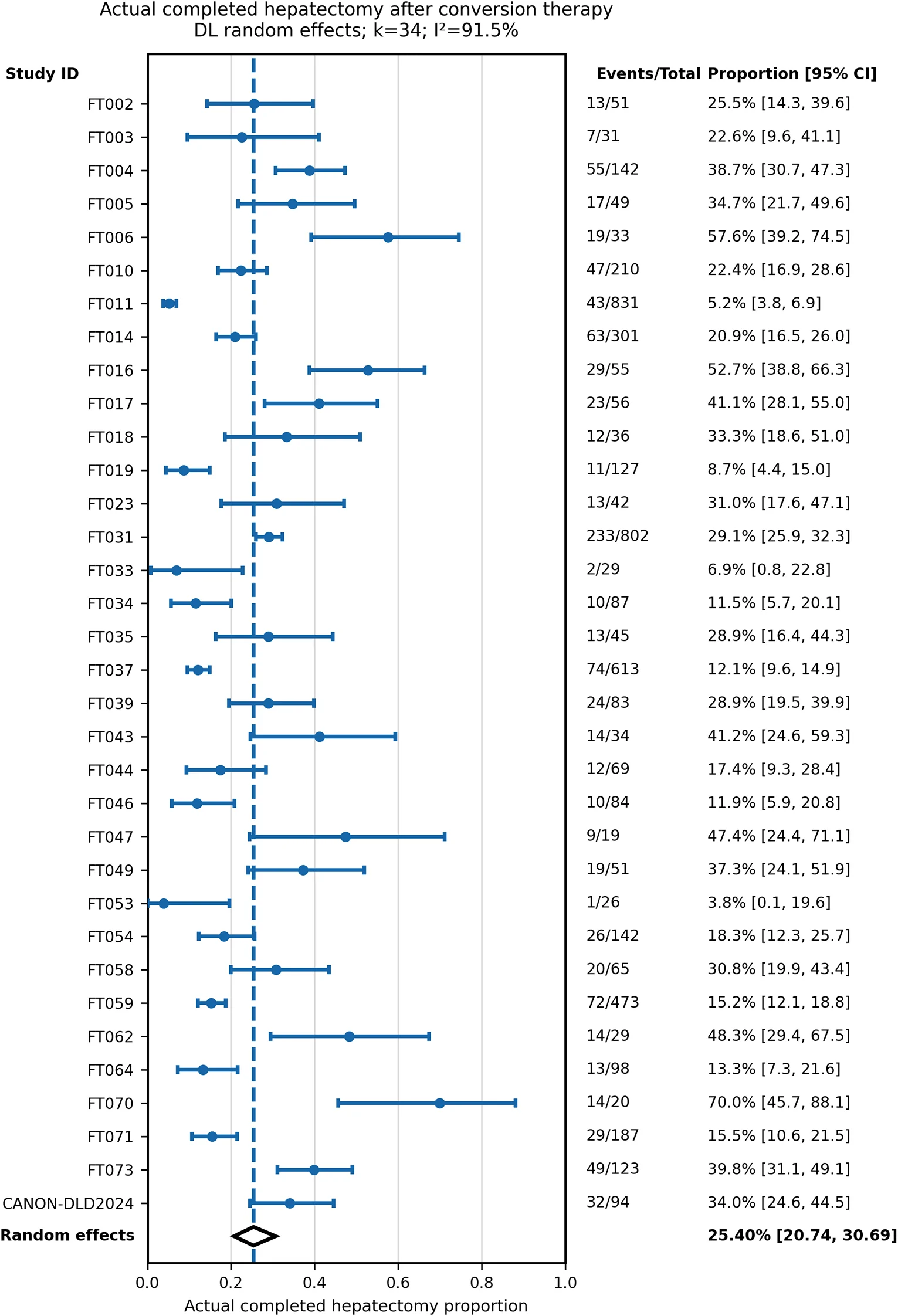
Random-effects forest plot of actual completed hepatectomy proportions in 34 studies. The pooled estimate was 25.40% (95% CI, 20.74%-30.69%).

Heterogeneity was substantial (I^2^=91.5%; tau^2^=0.5132), and the 95% prediction interval ranged from 7.16% to 60.04%. This dispersion is consistent with variation in tumor burden, macrovascular invasion, liver reserve, treatment composition, timing of surgical reassessment, and institutional thresholds for technical and oncological resectability.

### Subgroup and sensitivity analyses

Exploratory treatment-strategy subgroup analysis showed pooled rates of 28.16% (95% CI, 13.73%-49.11%; k=3; N = 279) for systemic therapy alone, 17.80% (95% CI, 11.44%-26.62%; k=11; N = 2,307) for locoregional therapy alone, and 30.15% (95% CI, 24.74%-36.17%; k=23; N = 1,938) for locoregional plus systemic therapy. The systemic-therapy-alone estimate was based on only three studies and had a wide confidence interval; it should therefore be regarded as particularly imprecise and exploratory. The omnibus meta-regression test was not statistically significant (Qm=5.17, df=2, P = 0.075), and these ecological comparisons do not establish equivalence, superiority, or treatment-specific efficacy (**Table 3**; **Figure 4**).

**Table 3:** Exploratory treatment-strategy subgroup analysis (mixed-effects meta-regression Qm=5.17, df=2, P = 0.075).

Strategy	k	N	Events	Pooled rate	95% CI	I^2^
Systemic therapy alone	3	279	64	28.16%	13.73%-49.11%	88.5%
Locoregional therapy alone	11	2,307	335	17.80%	11.44%-26.62%	92.9%
Locoregional plus systemic therapy	23	1,938	569	30.15%	24.74%-36.17%	84.1%

**Figure 4:**
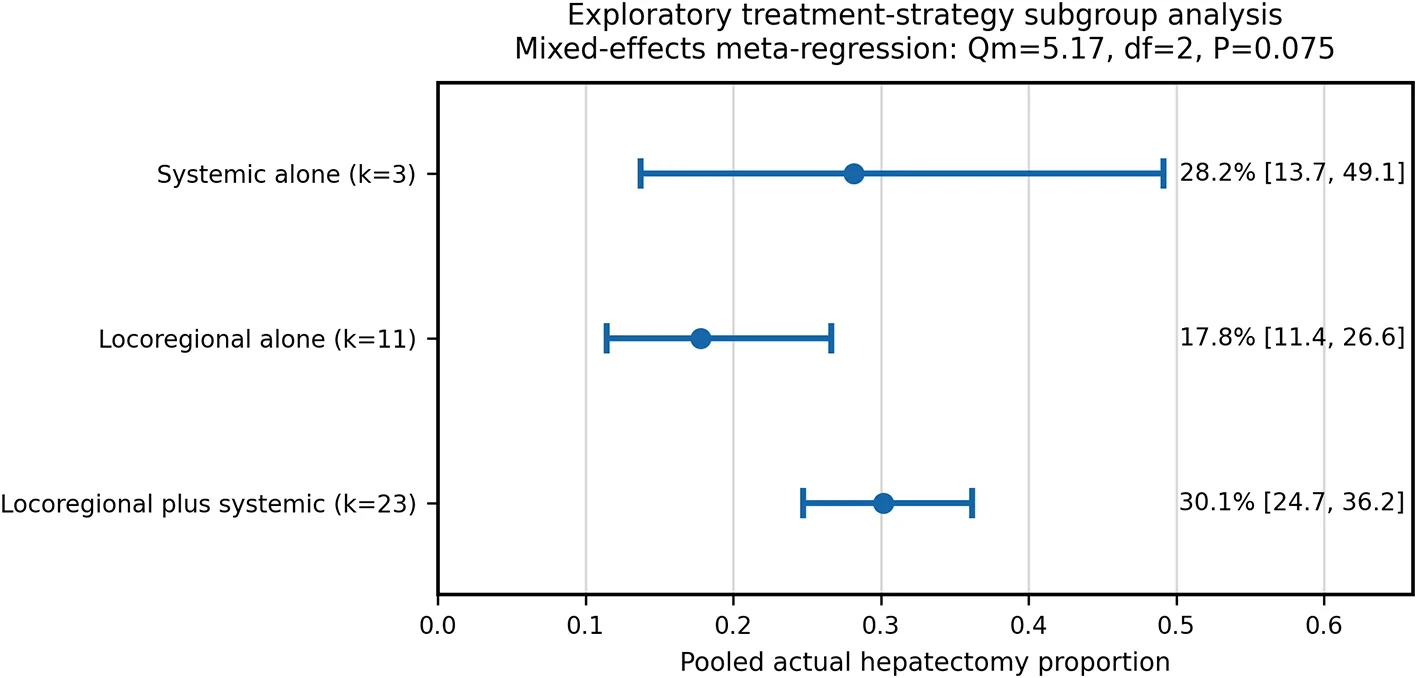
Exploratory treatment-strategy subgroup analysis using mutually exclusive classes. The omnibus meta-regression test was not statistically significant (Qm=5.17, df=2, P = 0.075); estimates should not be interpreted as causal comparisons.

Sensitivity analyses were consistent with the primary estimate. Including FT020 under a broader reported conversion-resection definition yielded 25.04% (95% CI, 20.47%-30.23%); restricting to contemporary modalities yielded 25.67% (95% CI, 20.70%-31.36%); and restricting to the pre-existing highest source-verification category (Evidence Tier A) yielded 25.58% (95% CI, 20.13%-31.92%).

Excluding historical iodine-131 FT058 yielded 25.24% (95% CI, 20.50%-30.65%), excluding historical high-dose TACE FT059 yielded 25.82% (95% CI, 20.95%-31.38%), and excluding the historical broad TACE-downstaging FT011 cohort yielded 26.64% (95% CI, 22.40%-31.36%) (**Supplementary Figure S1**).

Because the included publications spanned 1998-2026, publication year was additionally examined as a continuous moderator in a *post hoc* exploratory random-effects meta-regression. The slope was 0.0170 log-odds per year (95% CI, -0.0262 to 0.0602), corresponding to an odds ratio of 1.19 per decade (95% CI, 0.77-1.83; Qm=0.60, P = 0.440; residual tau^2^=0.5076). Thus, no clear linear study-level association between publication year and resection rate was detected. This analysis has limited power and publication year is only an ecological proxy for treatment era; it cannot isolate changes in regimens, imaging, patient selection, surgical practice, or reassessment thresholds (**Supplementary Table S7**).

### Small-study effects, sensitivity analysis, and heterogeneity diagnostics

Funnel plot inspection and Egger’s regression were used to assess small-study effects (**Figure 1**). Egger’s test did not indicate statistically significant asymmetry (P = 0.381). This does not exclude selective reporting in a highly heterogeneous observational literature, but it provided no evidence for a systematic funnel-plot asymmetry signal.

Endpoint-definition, evidence-tier, source-era, and historical-modality sensitivity analyses produced point estimates close to the primary result. Because funnel-based adjustment methods are unstable under substantial heterogeneity, no adjusted estimate was used to replace the prespecified primary model.

Leave-one-out sensitivity analysis showed stable results. Sequential omission of individual studies produced pooled rates of 24.52%-26.64% (**Figure 5**). Omitting the large historical Zhang YQ cohort produced the highest estimate and reduced heterogeneity, but no single-study omission changed the overall interpretation.

**Figure 5:**
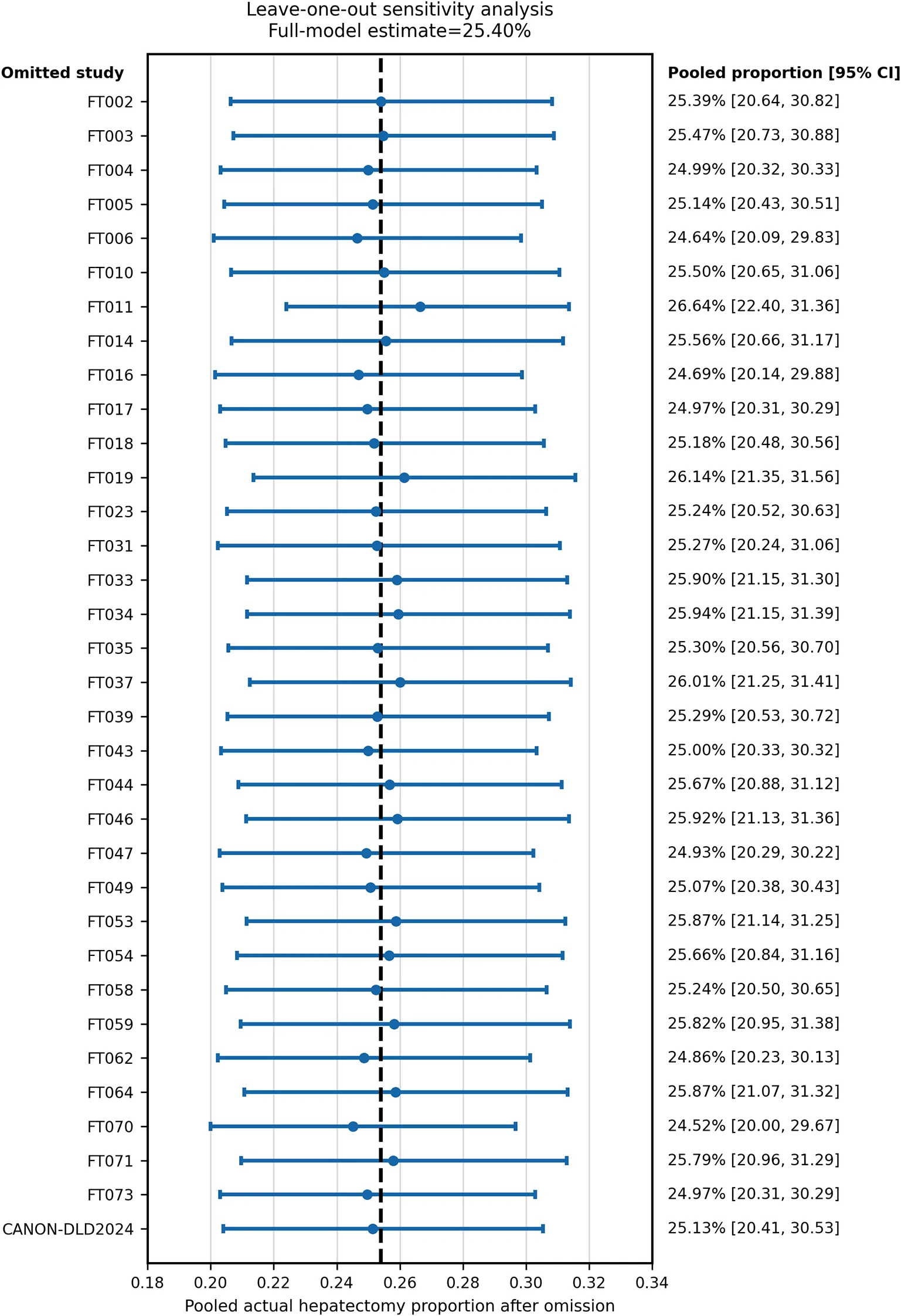
Leave-one-out sensitivity analysis. Sequential omission of individual studies produced pooled estimates of 24.52%-26.64%.

The exploratory treatment-strategy comparison was not statistically significant (Qm=5.17, df=2, P = 0.075). No study-level moderator result was used to support causal or treatment-ranking claims because aggregate-study associations are vulnerable to ecological bias and residual confounding.

### Secondary descriptive synthesis

Survival, recurrence, radiological response, pathological response, and postoperative outcomes were summarized descriptively across the 36 unique studies in **Supplementary Table S6**. These outcomes were not pooled because denominators, follow-up schedules, response criteria, and selection into surgery or pathology-evaluable subgroups differed substantially.

## Discussion

This systematic review found a crude aggregate completed-hepatectomy rate of 20.3% (1,042/5,137) and a random-effects modeled rate of 25.40% across 34 studies. The divergence is expected under marked between-study heterogeneity (I^2^=91.5%) because the random-effects model estimates an average study-level probability and does not weight studies solely by sample size. The prediction interval of 7.16%-60.04% further demonstrates that the modeled estimate is a cross-study benchmark rather than a fixed probability for an individual patient or center.

### Comparison with previous meta-analyses

The estimate is numerically consistent with, rather than contradictory to, previous syntheses. Pei et al. reported a 25% conversion rate for combined locoregional-systemic therapy; Li et al. reported 35% for lenvatinib plus an immune checkpoint inhibitor and locoregional therapy; Xu et al. reported 28% for TKI plus anti-PD-1 therapy and 33%-35% for triple therapy; and Hu et al. reported an overall conversion rate of 23%, with rates around 30% for ICI-TKI-TACE/HAIC combinations (5–8). Convergence is expected because many source cohorts overlap conceptually with the contemporary combination-therapy evidence base. The incremental contribution of the present review is that the estimand was restricted to completed liver resection among all treated patients across regimen classes. Radiological conversion, eligibility for surgery, transplantation, ablation, and post-resection-only cohorts were not allowed to inflate the numerator or narrow the denominator.

Clinical interpretation should therefore focus on care pathways rather than ranking regimens. Contemporary HCC guidance emphasizes multidisciplinary evaluation, while Chinese conversion-therapy consensus statements recommend individualized combinations of systemic and locoregional treatment, repeated response and liver-reserve assessment, and timely surgical reassessment after successful conversion (2, 55, 56). The approximately one-quarter benchmark can inform patient counselling and service planning, but it should not be used as a treatment-specific efficacy claim. Decisions to proceed to surgery require simultaneous assessment of technical resectability, oncological benefit, future liver remnant, liver function, treatment toxicity, and patient preference.

The heterogeneity is clinically coherent. Tumor size and multiplicity, macrovascular invasion, extrahepatic disease restrictions, liver function, etiology, response criteria, conversion regimen, treatment duration, reassessment interval, and institutional surgical thresholds varied substantially. Access to high-volume interventional and hepatobiliary teams may further influence whether a radiological response culminates in resection. Treatment-strategy estimates overlapped widely and the omnibus comparison was not statistically significant; between-study confounding, arm selection, sparse subgroups, and substantial residual heterogeneity preclude comparative causal interpretation (2, 55, 56). In particular, the systemic-therapy-alone subgroup contained only three studies and yielded a wide confidence interval, so it should not be used to infer equivalence or superiority relative to locoregional-containing strategies.

The historical Zhang YQ 2016 cohort and the Su 2026 prediction-model report were retained for distinct, explicit reasons. Zhang YQ 2016 enrolled patients with baseline unresectable HCC who received TACE and reported both an all-treated denominator and completed liver resections. We treated it as a historical broad downstaging cohort, not as evidence directly comparable with contemporary protocolized conversion regimens; its broad initial-treatment context and low resection proportion were addressed by a separate historical-cohort sensitivity analysis. Su 2026 was eligible because its underlying clinical cohort comprised all treated patients with unresectable HCC and reported actual radical resection events (47/210). Its nomogram-development purpose does not alter the denominator or event definition for the descriptive resection-rate estimand, but its internally validated predictor findings were not pooled or interpreted as treatment effects.

Source-level adjudication produced several transparent corrections. FT020 was excluded from the primary endpoint because 3/26 represented attainment of surgical-treatment criteria without explicit completed hepatectomy. GUIDANCE001 (FT031) remained 233/802 after review of the original report and erratum; the erratum did not alter hepatectomy counts. FT071 remained the canonical Zhongshan-family report (29/187). Final re-extraction also corrected Du Y 2023 to 2/29, Li B 2021 to 24/83, Gan LJ 2023 to 13/98, and Cao YJ 2023 to 14/20. These corrections changed the aggregate denominator and event count but did not change the clinical interpretation.

Small-study-effect assessment reinforces the need for restraint. Egger’s test was not statistically significant (P = 0.381), but selective reporting cannot be ruled out because referral patterns, selective enrollment, center expertise, and preferential publication of high-response cohorts may not produce a detectable funnel-plot signal.

Secondary survival, recurrence, pathological-response, and postoperative outcomes were not pooled because most were reported among selected patients who reached surgery, achieved radiological response, or had evaluable pathology. Those outcomes answer a different question from the baseline probability of reaching resection and are especially vulnerable to selection, immortal-time, and surgical-candidacy biases. Future studies should define a common time zero, report reasons for non-resection, specify reassessment timing and criteria, and provide both all-treated and post-resection outcomes.

Several limitations remain. Most studies were nonrandomized and retrospective; definitions of unresectability and conversion intent were inconsistent; and aggregate data prevented adjustment for tumor burden, vascular invasion, liver reserve, response, or surgical candidacy. Thirty-two of the 34 primary-analysis studies were conducted in China, predominantly in centers experienced in locoregional therapy and hepatobiliary surgery, limiting international generalizability. Although the canonical-report hierarchy reduced known double counting, residual cohort overlap cannot be completely excluded when patient-level identifiers are unavailable. The hierarchy was transparently reported as a *post hoc* report-selection decision. The overall certainty of evidence was rated very low, substantial heterogeneity remained, and undetected selective reporting cannot be excluded.

The evidence base also spans almost three decades, from 1998 to 2026, during which systemic therapy, locoregional techniques, imaging, perioperative care, surgical selection, and multidisciplinary reassessment changed substantially. The exploratory publication-year meta-regression did not detect a statistically significant linear temporal association (OR per decade 1.19, 95% CI 0.77-1.83; P = 0.440), but this finding does not remove treatment-era confounding: the analysis is study-level, has limited power, and publication year is only an imperfect proxy for multiple simultaneous changes in clinical practice. The primary pooled estimate should therefore be interpreted as a cross-era benchmark and not as the expected probability under any specific contemporary regimen.

A further methodological limitation is that the primary quantitative objective changed after preliminary data extraction had begun. The original PROSPERO registration specified predictors of successful conversion as the primary objective; heterogeneous predictor definitions and analytical frameworks precluded a defensible predictor meta-analysis, and no predictor meta-analysis was performed. The protocol was subsequently amended on May 1, 2026, to specify completed liver resection among all treated patients as the primary quantitative outcome. Although the amendment was documented before construction of the final outcome-specific dataset and final quantitative synthesis, it occurred after preliminary extraction had started and therefore introduces a potential risk of *post hoc* outcome specification and selective emphasis. The pooled resection-rate analysis should consequently be interpreted as a transparently amended primary analysis rather than the originally registered primary outcome.

## Conclusion

In very-low-certainty study-level evidence, approximately one quarter of patients with initially unresectable or baseline unresectable HCC underwent completed liver resection after conversion or downstaging therapy when a consistently applied all-treated denominator was used. The estimate is a conservative benchmark for conversion-to-surgery pathways, not evidence that any regimen is superior or that conversion therapy independently improves survival. Its wide prediction interval, residual heterogeneity, predominantly Chinese evidence base, and possibility of selective reporting support standardized prospective reporting and repeated multidisciplinary surgical reassessment.

## Data Availability

Study-level data were extracted from published reports. Zenodo repository: https://doi.org/10.5281/zenodo.21976635 Accession/DOI: 10.5281/zenodo.21976635 (57). The repository contains the final 34-study analytic dataset, data dictionary, reproducible verification code for the primary and treatment-era analyses, analysis summary, and supporting output files. No individual participant-level data are included.

## Supplementary material

Supplementary Figure 1 Sensitivity analysis comparing the endpoint-compatible primary model with broader endpoint, contemporary-modality, evidence-tier, and historical‑study scenarios.

Supplementary Figure 2 **(A)** Newcastle-Ottawa Scale domain-level judgments for 14 comparative nonrandomized studies. **(B)** JBI case-series checklist item-level judgments for 19 noncomparative primary-analysis studies. **(C)** RoB 2 domain-level judgments for the randomized FT059 study; the overall judgment was high risk.
