# Supplementary material for: Resection rates after conversion therapy for initially unresectable hepatocellular carcinoma: a systematic review and meta-analysis
Source: Front Oncol. 2026 Oct 1;16:1898480. doi: 10.3389/fonc.2026.1898480 (PMC13668533; doi:10.3389/fonc.2026.1898480)
Supplement: Supplementary file 1 [file Supplementaryfile1.docx]

**PRISMA 2020 Checklist**

**Resection Rates after Conversion Therapy for Initially Unresectable Hepatocellular Carcinoma: A Systematic Review and Meta-analysis**

Checklist based on the PRISMA 2020 statement (Page et al., BMJ 2021;372:n71). Locations use stable section, table, and figure labels rather than pagination that may change during production. The PRISMA 2020 checklist template is distributed under the Creative Commons Attribution 4.0 International (CC BY 4.0) license.

| **Section/topic** | **Item** | **Checklist item** | **Location where reported** |
| --- | --- | --- | --- |
| TITLE | 1 | Identify the report as a systematic review. | Title |
| ABSTRACT | 2 | See the PRISMA 2020 for Abstracts checklist. | Structured Abstract |
| INTRODUCTION | 3 | Describe the rationale for the review in the context of existing knowledge. | Introduction—paragraphs 1-5 |
| INTRODUCTION | 4 | Provide an explicit statement of the objective(s) or question(s) the review addresses. | End of Introduction |
| METHODS | 5 | Specify the inclusion and exclusion criteria for the review and how studies were grouped for syntheses. | Methods—Search strategy and eligibility criteria (explicit PICOS framework) |
| METHODS | 6 | Specify all databases, registers, websites, organisations, reference lists, and other sources searched or consulted, and the date each source was last searched. | Methods; Supplementary Table S2 |
| METHODS | 7 | Present the full search strategies for all databases, registers, and websites, including filters and limits used. | Supplementary Table S2 |
| METHODS | 8 | Specify the methods used to decide whether a study met the inclusion criteria, including reviewer independence and any automation tools used. | Methods—Study selection and data extraction |
| METHODS | 9 | Specify the methods used to collect data from reports, including reviewer independence and any processes for obtaining or confirming data. | Methods—Study selection and data extraction |
| METHODS | 10a | List and define all outcomes for which data were sought and specify whether all compatible results were sought. | Methods—Outcomes and statistical analysis |
| METHODS | 10b | List and define all other variables for which data were sought and describe assumptions about missing or unclear information. | Methods—Study selection and data extraction |
| METHODS | 11 | Specify the methods used to assess risk of bias in included studies, including the tool(s), reviewer independence, and any automation tools. | Methods—Risk of bias assessment; Supplementary Table S3 |
| METHODS | 12 | Specify for each outcome the effect measure(s) used in the synthesis or presentation of results. | Methods—Outcomes and statistical analysis |
| METHODS | 13a | Describe the processes used to decide which studies were eligible for each synthesis. | Methods—Eligibility; data extraction; subgroup definitions |
| METHODS | 13b | Describe any methods required to prepare data for presentation or synthesis. | Methods—Study selection and data extraction |
| METHODS | 13c | Describe methods used to tabulate or visually display results of individual studies and syntheses. | Methods; Tables 1-3; Figures 1 and 3-5 |
| METHODS | 13d | Describe methods used to synthesize results and justify choices; if meta-analysis was performed, describe model, heterogeneity assessment, and software. | Methods—Outcomes and statistical analysis; software and reproducibility statement |
| METHODS | 13e | Describe methods used to explore possible causes of heterogeneity among study results. | Methods—treatment-strategy and publication-year meta-regression; Table 3; Figure 4; Supplementary Table S7 |
| METHODS | 13f | Describe sensitivity analyses conducted to assess robustness of synthesized results. | Methods—Sensitivity analyses; Figure 5; Supplementary Figure S1 |
| METHODS | 14 | Describe methods used to assess risk of bias due to missing results in a synthesis. | Methods—Small-study effects; Figure 1 |
| METHODS | 15 | Describe methods used to assess certainty in the body of evidence for an outcome. | Methods—Certainty of evidence; Supplementary Table S4 |
| RESULTS | 16a | Describe the results of the search and selection process, ideally using a flow diagram. | Results—Study selection; Figure 2 |
| RESULTS | 16b | Cite studies that might appear to meet inclusion criteria but were excluded, and explain why they were excluded. | Supplementary Table S5 |
| RESULTS | 17 | Cite each included study and present its characteristics. | Table 1; Supplementary Tables S1 and S6 |
| RESULTS | 18 | Present assessments of risk of bias for each included study. | Results; Supplementary Table S3; Supplementary Figures S2A-S2C |
| RESULTS | 19 | For all outcomes, present results for each study and summary statistics as appropriate. | Table 1; Figure 3; Supplementary Table S6 |
| RESULTS | 20a | For each synthesis, briefly summarise the characteristics and risk of bias among contributing studies. | Results—Study characteristics and risk of bias |
| RESULTS | 20b | Present results of all statistical syntheses, including precision and heterogeneity; for comparisons, present the direction of effect. | Results; Tables 2-3; Figures 3 and 4 |
| RESULTS | 20c | Present results of investigations of possible causes of heterogeneity. | Results—treatment-strategy and publication-year meta-regression; Table 3; Figure 4; Supplementary Table S7 |
| RESULTS | 20d | Present results of all sensitivity analyses conducted to assess robustness. | Results—Sensitivity analyses; Figure 5; Supplementary Figure S1 |
| RESULTS | 21 | Present assessments of risk of bias due to missing results for each synthesis assessed. | Results—Small-study effects; Figure 1 |
| RESULTS | 22 | Present assessments of certainty in the body of evidence for each outcome assessed. | Results—Certainty; Supplementary Table S4 |
| DISCUSSION | 23a | Provide a general interpretation of the results in the context of other evidence. | Discussion—Comparison with previous meta-analyses |
| DISCUSSION | 23b | Discuss limitations of the evidence included in the review. | Discussion—evidence limitations, treatment-era span, generalizability, and heterogeneity |
| DISCUSSION | 23c | Discuss limitations of the review processes used. | Discussion—review-process limitations, including protocol amendment and post hoc report-selection hierarchy |
| DISCUSSION | 23d | Discuss implications of the results for practice, policy, and future research. | Discussion—Clinical interpretation; Conclusion |
| OTHER | 24a | Provide registration information, including register name and registration number, or state that the review was not registered. | Methods—Study design and registration |
| OTHER | 24b | Indicate where the review protocol can be accessed, or state that a protocol was not prepared. | Methods—PROSPERO record |
| OTHER | 24c | Describe and explain any amendments to information provided at registration or in the protocol. | Methods—Study design and registration; Discussion—Limitations; Supplementary Table S2A |
| OTHER | 25 | Describe sources of financial or non-financial support and the role of funders or sponsors. | Funding statement |
| OTHER | 26 | Declare competing interests of review authors. | Conflict of interest statement |
| OTHER | 27 | Report which materials are publicly available and where they can be found. | Data availability statement; Zenodo DOI 10.5281/zenodo.21976635 |
