# Supplementary material for: Resection rates after conversion therapy for initially unresectable hepatocellular carcinoma: a systematic review and meta-analysis
Source: Front Oncol. 2026 Oct 1;16:1898480. doi: 10.3389/fonc.2026.1898480 (PMC13668533; doi:10.3389/fonc.2026.1898480)
Supplement: Supplementary file 2 [file Table1.docx]

Supplementary Table S1A. Characteristics of studies included in the endpoint-compatible primary quantitative synthesis

Endpoint: actual liver resection/hepatectomy/salvage liver resection after conversion or downstaging therapy. Liver transplantation, ablation, RFA/SBRT, resection eligibility, and radiologic conversion alone were not counted as resection events.

| **No.** | **Study ID** | **Citation** | **Strategy** | **N** | **Resection n** | **Rate** | **Tier** | Context | **Source identifier** |
| --- | --- | --- | --- | --- | --- | --- | --- | --- | --- |
| 1 | FT002 | Lu YC et al., 2024, Chinese Journal of Hepatobiliary Surgery [in Chinese] | Locoregional plus systemic | 51 | 13 | 25.5% | A | — | DOI 10.3760/cma.j.cn113884-20240719-00219 |
| 2 | FT003 | Bai Z et al., 2024, British Journal of Hospital Medicine | Locoregional plus systemic | 31 | 7 | 22.6% | A | — | DOI 10.12968/hmed.2024.0159 |
| 3 | FT004 | Zhang X et al., 2026, Signal Transduction and Targeted Therapy | Locoregional alone / Locoregional plus systemic | 142 | 55 | 38.7% | A | — | DOI 10.1038/s41392-025-02498-z |
| 4 | FT005 | Pan X et al., 2023, Journal of Hepatocellular Carcinoma | Locoregional plus systemic | 49 | 17 | 34.7% | A | — | DOI 10.2147/JHC.S404500 |
| 5 | FT006 | He C et al., 2022, Frontiers in Oncology | Locoregional alone | 33 | 19 | 57.6% | A | — | DOI 10.3389/fonc.2022.923566 |
| 6 | FT010 | Su Y et al., 2026, European Journal of Radiology | Locoregional plus systemic | 210 | 47 | 22.4% | A | — | DOI 10.1016/j.ejrad.2026.112724 |
| 7 | FT011 | Zhang YQ et al., 2016, The Oncologist | Locoregional alone | 831 | 43 | 5.2% | A | Broad TACE | DOI 10.1634/theoncologist.2016-0094 |

| **No.** | **Study ID** | **Citation** | **Strategy** | **N** | **Resection n** | **Rate** | **Tier** | Context | **Source identifier** |
| --- | --- | --- | --- | --- | --- | --- | --- | --- | --- |
| 8 | FT014 | Lin SR et al., 2025, Liver Cancer | Locoregional alone / Locoregional plus systemic | 301 | 63 | 20.9% | A | — | DOI 10.1159/000547725 |
| 9 | FT016 | Wu XK et al., 2024, eClinicalMedicine | Locoregional plus systemic | 55 | 29 | 52.7% | A | — | DOI 10.1016/j.eclinm.2023.102367 |
| 10 | FT017 | Zhang W et al., 2023, Journal for ImmunoTherapy of Cancer | Systemic alone | 56 | 23 | 41.1% | B | — | DOI 10.1136/jitc-2023-007366 |
| 11 | FT018 | Wang L et al., 2023, Frontiers in Oncology | Systemic alone | 36 | 12 | 33.3% | B | — | DOI 10.3389/fonc.2023.1115109 |
| 12 | FT019 | Regnault H et al., 2024, Hepatology Communications | Locoregional alone | 127 | 11 | 8.7% | A | — | DOI 10.1097/HC9.0000000000000475 |
| 13 | FT023 | Zhang YC et al., 2023, Chinese Journal of Hepatobiliary Surgery [in Chinese] | Locoregional plus systemic | 42 | 13 | 31.0% | A | — | DOI 10.3760/cma.j.cn113884-20230310-00074 |
| 14 | FT031 | Yang DL et al., 2025, Hepatology (GUIDANCE001) | Locoregional alone / Locoregional plus systemic | 802 | 233 | 29.1% | C | — | DOI 10.1097/HEP.0000000000001229; Erratum DOI 10.1097/HEP.0000000000001401 |
| 15 | FT033 | Du Y et al., 2023, Herald of Medicine [in Chinese] | Locoregional alone / Locoregional plus systemic | 29 | 2 | 6.9% | B | — | DOI 10.3870/j.issn.1004-0781.2023.11.024 |
| 16 | FT034 | Li J et al., 2023, Frontiers in Immunology | Locoregional plus systemic | 87 | 10 | 11.5% | A | — | DOI 10.3389/fimmu.2023.1188308 |

| **No.** | **Study ID** | **Citation** | **Strategy** | **N** | **Resection n** | **Rate** | **Tier** | Context | **Source identifier** |
| --- | --- | --- | --- | --- | --- | --- | --- | --- | --- |
| 17 | FT035 | He ZL et al., 2026, Minimally Invasive Medicine [in Chinese] | Locoregional plus systemic | 45 | 13 | 28.9% | A | — | Minimally Invasive Medicine. 2026;21(1):40-45. DOI 10.11864/j.issn.1673.2026.01.06 |
| 18 | FT037 | Piao M et al., 2025, Liver Cancer | Systemic-based; variable locoregional component (not subgrouped) | 613 | 74 | 12.1% | A | — | DOI 10.1159/000543994 |
| 19 | FT039 | Li B et al., 2021, Annals of Surgery Open | Locoregional alone | 83 | 24 | 28.9% | A | — | DOI 10.1097/AS9.0000000000000057 |
| 20 | FT043 | Zhang J et al., 2021, Frontiers in Oncology | Locoregional plus systemic | 34 | 14 | 41.2% | A | — | DOI 10.3389/fonc.2021.729764 |
| 21 | FT044 | Yang YG et al., 2023, Chinese Journal of Hepatic Surgery (Electronic Edition) [in Chinese] | Locoregional plus systemic | 69 | 12 | 17.4% | A | — | DOI 10.3877/cma.j.issn.2095-3232.2023.01.011 |
| 22 | FT046 | Zeng ST et al., 2025, Chinese Journal of Hepatic Surgery (Electronic Edition) [in Chinese] | Locoregional plus systemic | 84 | 10 | 11.9% | A | — | DOI 10.3877/cma.j.issn.2095-3232.2025041 |
| 23 | FT047 | Yalikun K et al., 2025, BMC Cancer | Locoregional plus systemic | 19 | 9 | 47.4% | A | — | DOI 10.1186/s12885-025-14250-5 |
| 24 | FT049 | Qu WF et al., 2022, BJS Open | Locoregional plus systemic | 51 | 19 | 37.3% | A | — | DOI 10.1093/bjsopen/zrac114 |
| 25 | FT053 | Son SY et al., 2024, Cancers | Locoregional alone | 26 | 1 | 3.8% | A | — | DOI 10.3390/cancers16173024 |

| **No.** | **Study ID** | **Citation** | **Strategy** | **N** | **Resection n** | **Rate** | **Tier** | Context | **Source identifier** |
| --- | --- | --- | --- | --- | --- | --- | --- | --- | --- |
| 26 | FT054 | Chen S et al., 2022, Journal of Cancer Research and Clinical Oncology | Locoregional plus systemic | 142 | 26 | 18.3% | A | — | DOI 10.1007/s00432-021-03767-4 |
| 27 | FT058 | Zeng ZC et al., 1998, Journal of Cancer Research and Clinical Oncology | Locoregional alone | 65 | 20 | 30.8% | A | Pre-modern | DOI 10.1007/s004320050166 |
| 28 | FT059 | Chen MS et al., 2002, World Journal of Gastroenterology | Locoregional alone | 473 | 72 | 15.2% | A | Pre-modern | DOI 10.3748/wjg.v8.i1.74 |
| 29 | FT062 | Liu D et al., 2023, Neoplasma | Locoregional plus systemic | 29 | 14 | 48.3% | A | — | DOI 10.4149/neo_2023_230806N413 |
| 30 | FT064 | Gan LJ et al., 2023, Journal of Hepatocellular Carcinoma | Locoregional plus systemic | 98 | 13 | 13.3% | A | — | DOI 10.2147/JHC.S404675 |
| 31 | FT070 | Cao YJ et al., 2023, Clinical Oncology Journal [in Chinese] | Locoregional plus systemic | 20 | 14 | 70.0% | A | — | Clinical Oncology Journal. 2023;28(6):481-487; no DOI displayed in the published PDF. |
| 32 | FT071 | Xu B et al., 2022, Frontiers in Immunology | Systemic alone | 187 | 29 | 15.5% | C | — | DOI 10.3389/fimmu.2022.1016736 |
| 33 | FT073 | Zhou Y et al., 2025, BioScience Trends | Locoregional plus systemic, with/without radiotherapy | 123 | 49 | 39.8% | A | — | DOI 10.5582/bst.2025.01170 |
| 34 | Li X 2024 | Li X et al., 2024, Digestive and Liver Disease | Locoregional plus systemic | 94 | 32 | 34.0% | A | — | DOI 10.1016/j.dld.2023.11.027 |

Supplementary Table S1B. Study-level source, endpoint, and canonical-report adjudications affecting the primary synthesis

| **Study ID** | **Issue** | **Evidence reviewed** | **Final decision** | **Primary model effect** | **Status** |
| --- | --- | --- | --- | --- | --- |
| FT020 | Reported conversion-resection rate without explicit completed hepatectomy | Full Chinese article | Qualitative only; exclude from primary endpoint | No addition of 3/26 | Closed |
| FT031 | Original report plus formal erratum | Hepatology article and erratum | Include 233/802 | Included | Closed |
| FT071 | Overlapping Zhongshan report family | Canonical-report assessment | Include 29/187 | Included | Closed |
| FT011 | Historical broad TACE-downstaging cohort | All-treated denominator and completed resection count; historical treatment context | Retain in primary model; evaluate in historical-cohort sensitivity analysis | Historical sensitivity | Closed |
| FT010 | Prediction-model report | Underlying all-treated cohort with 47/210 actual radical resections | Retain for primary resection-rate estimand; do not pool predictor findings | No predictor meta-analysis | Closed |
| FT033 | Ablation incorrectly counted as hepatectomy in an earlier extraction | Full Chinese article | Include 2/29 completed hepatectomies; exclude one ablation | Corrected primary event | Closed |
| FT039 | Earlier extraction used a restricted denominator | Full article and treatment-arm results | Include 24/83 (20/41 plus 4/42 actual hepatic resections) | Corrected N/events | Closed |
| FT064 | Successful conversion included two RFA procedures | Full article results | Include 13/98 actual hepatectomies; exclude two RFA procedures | Corrected primary event | Closed |
| FT070 | Earlier extraction omitted treated participants/resections | Full Chinese article | Include 14/20 completed R0 resections | Corrected N/events | Closed |
| Li X 2024 / Li X 2023 | Linked companion reports from the same 94-patient cohort | Same institution, recruitment period, denominator, regimen, and linked authorship across the 2023 Frontiers in Oncology and 2024 Digestive and Liver Disease reports | Count once; use Li X 2024 as the canonical primary report and retain Li X 2023 for secondary context | One primary study-level row (94/32) | Closed |
| Wei 2021 | Potential network overlap with a larger multicenter cohort could not be excluded | Center/recruitment/authorship/regimen overlap assessment from the review audit | Retain descriptively; exclude from primary pooling to avoid possible double counting | No contribution to primary pooled denominator | Closed |

Abbreviations: HAIC, hepatic arterial infusion chemotherapy; HCC, hepatocellular carcinoma; ICI, immune checkpoint inhibitor; PD-1, programmed cell death protein 1; PSM, propensity-score matching; PVE, portal vein embolization; RFA, radiofrequency ablation; SBRT, stereotactic body radiotherapy; SIRT, selective internal radiation therapy; TACE, transarterial chemoembolization; TKI, tyrosine kinase inhibitor.

Notes: N is the all-treated denominator used for the primary synthesis, and the event is actual completed hepatectomy. Source identifiers are provided for traceability. All endpoint, source, overlap, and extraction adjudications affecting the primary synthesis are summarized in Supplementary Table S1B. The Context column identifies cohorts evaluated in context-specific historical-treatment sensitivity analyses: FT011 was a broad TACE cohort with historical treatment context, whereas FT058 and FT059 used pre-modern modalities; an em dash indicates no such designation.

Evidence-tier note. Evidence-tier labels in Supplementary Table S1A are source-verification categories from the review audit and are not risk-of-bias or study-design grades. Tier A denotes the highest source-verification category and was used only for a sensitivity analysis; formal risk-of-bias assessment was performed separately with NOS, JBI, or RoB 2 as appropriate.

Treatment-strategy definitions: systemic therapy alone = systemic agents without planned locoregional therapy; locoregional therapy alone = TACE, HAIC, SIRT/radioembolization, portal vein embolization-based strategies, or other regional approaches without systemic agents; locoregional plus systemic therapy = a named combination of regional and systemic treatment, including regimens with radiotherapy. Multiarm studies were separated only when arms belonged to different classes; same-class arms were combined before meta-analysis. Piao M 2025 was retained overall but excluded from strategy subgroups because variable locoregional components could not be assigned consistently. The strategy analysis comprised 37 independent class-specific units from 33 parent studies.

Supplementary Table S2. Database-specific search strategies recorded in the review materials

Note: Searches were conducted from database inception to April 10, 2026. Database-specific search strategies and counts are reported as documented in the review records. The strings, platforms, dates, limits, and pre-deduplication record counts below are therefore presented as recorded in the review materials. The reported counts are PubMed/MEDLINE (n=149), Web of Science Core Collection (n=155), Cochrane Library/CENTRAL (n=167), Embase (n=267), Wanfang Data (n=215), and CNKI (n=3), totaling 956 records and matching the PRISMA arithmetic.

| Database / source | Records identified before deduplication | Platform / field | Search strategy recorded in the review materials | Limits and date searched |
| --- | --- | --- | --- | --- |
| PubMed/MEDLINE | 149 | Title/Abstract and MeSH | (("Carcinoma, Hepatocellular"[Mesh] OR "hepatocellular carcinoma"[tiab] OR "hepatocellular cancer"[tiab] OR "liver cancer"[tiab] OR HCC[tiab]) AND (unresectable[tiab] OR "initially unresectable"[tiab] OR "locally advanced"[tiab] OR advanced[tiab]) AND ("conversion therapy"[tiab] OR downstaging[tiab] OR "conversion surgery"[tiab] OR "salvage surgery"[tiab] OR resection[tiab] OR hepatectomy[tiab]) AND (immunotherapy[tiab] OR "immune checkpoint inhibitor"[tiab] OR PD-1[tiab] OR PD-L1[tiab] OR lenvatinib[tiab] OR sorafenib[tiab] OR "targeted therapy"[tiab] OR TACE[tiab] OR HAIC[tiab])) | From database inception to 10 April 2026. No language restriction was applied at the search stage; animal-only studies were excluded during screening when identifiable. |
| Web of Science Core Collection | 155 | Topic search (TS) | TS=(("hepatocellular carcinoma" OR "hepatocellular cancer" OR "liver cancer" OR HCC) AND (unresectable OR "initially unresectable" OR "locally advanced" OR advanced) AND ("conversion therapy" OR downstaging OR "conversion surgery" OR "salvage surgery" OR resection OR hepatectomy) AND (immunotherapy OR "immune checkpoint inhibitor" OR PD-1 OR PD-L1 OR lenvatinib OR sorafenib OR "targeted therapy" OR TACE OR HAIC)) | From database inception to 10 April 2026. Document-type and language restrictions were not applied before screening. |
| Cochrane Library / CENTRAL | 167 | Title, abstract, keyword and MeSH | ([mh "Carcinoma, Hepatocellular"] OR "hepatocellular carcinoma" OR "hepatocellular cancer" OR "liver cancer" OR HCC) AND (unresectable OR "initially unresectable" OR "locally advanced" OR advanced) AND ("conversion therapy" OR downstaging OR "conversion surgery" OR "salvage surgery" OR resection OR hepatectomy OR TACE OR HAIC OR immunotherapy OR lenvatinib OR sorafenib) | From database inception to 10 April 2026. Trials and trial records were screened for extractable published data. |
| Embase | 267 | Title/abstract/keyword and Emtree terms | ('hepatocellular carcinoma'/exp OR 'hepatocellular carcinoma':ti,ab,kw OR 'hepatocellular cancer':ti,ab,kw OR 'liver cancer':ti,ab,kw OR HCC:ti,ab,kw) AND (unresectable:ti,ab,kw OR 'initially unresectable':ti,ab,kw OR 'locally advanced':ti,ab,kw OR advanced:ti,ab,kw) AND ('conversion therapy':ti,ab,kw OR downstaging:ti,ab,kw OR 'conversion surgery':ti,ab,kw OR 'salvage surgery':ti,ab,kw OR resection:ti,ab,kw OR hepatectomy:ti,ab,kw) AND (immunotherapy:ti,ab,kw OR 'immune checkpoint inhibitor':ti,ab,kw OR 'pd 1':ti,ab,kw OR 'pd l1':ti,ab,kw OR lenvatinib:ti,ab,kw OR sorafenib:ti,ab,kw OR 'targeted therapy':ti,ab,kw OR TACE:ti,ab,kw OR HAIC:ti,ab,kw) | From database inception to 10 April 2026. No language restriction was applied at the search stage. |
| Wanfang Data | 215 | Subject / title / abstract | Chinese database search: hepatocellular carcinoma AND unresectable/initially unresectable AND conversion/downstaging therapy AND liver resection/hepatectomy AND systemic or locoregional treatment (Chinese-language search terms translated into English). | From database inception to 10 April 2026. Chinese-language records were screened using the same eligibility criteria as English-language records. |
| CNKI | 3 | Subject / title / abstract | Chinese database search: hepatocellular carcinoma AND unresectable/initially unresectable AND conversion/downstaging therapy AND liver resection/hepatectomy AND systemic or locoregional treatment (Chinese-language search terms translated into English). | From database inception to 10 April 2026. Chinese-language records were screened using the same eligibility criteria as English-language records. |

**Supplementary Table S2A. Review chronology and protocol amendment**

| **Milestone** | **Date / range** | **Documented status** |
| --- | --- | --- |
| PROSPERO Version 1.0 published | April 5, 2026 | Searching/study identification had commenced but was incomplete; formal screening, data extraction, risk-of-bias assessment, and quantitative synthesis had not commenced. |
| Database searches completed | April 10, 2026 | Database searching was complete. |
| Preliminary review conduct | April 10 to May 1, 2026 | Preliminary screening, full-text assessment, data extraction, and risk-of-bias assessment were undertaken. |
| PROSPERO Version 2.0 published | May 1, 2026 | Primary outcome amended to actual completed liver resection among all treated patients; no predictor meta-analysis was undertaken. |
| Final outcome-specific dataset and synthesis | After May 1, 2026 | Study-level dataset, analytic decisions, and quantitative synthesis completed using the amended outcome definition. |

Supplementary Table S3. Design-specific domain-level risk-of-bias assessment of the 34 primary-synthesis studies

Note. Two reviewers (X.O. and J.L.) independently reassessed the 34 primary-synthesis studies using one design-appropriate tool, recorded supporting source information and reasons for each domain judgment, and compared their assessments only after independent completion. Discrepancies were resolved by source re-examination and consensus; unresolved disagreements were adjudicated by P.C. Comparative nonrandomized cohorts were evaluated using the Newcastle-Ottawa Scale (NOS), single-arm/noncomparative cohorts using the JBI case-series checklist, and FT059 using RoB 2. Reports were not scored with more than one tool, and cross-tool numerical totals were not combined.

Supplementary Table S3A. Newcastle-Ottawa Scale domain assessment for comparative nonrandomized cohorts

| **No.** | **Study ID** | **S1** | **S2** | **S3** | **S4** | **C1** | **C2** | **O1** | **O2** | **O3** | **Total** | **Interpretation** |
| --- | --- | --- | --- | --- | --- | --- | --- | --- | --- | --- | --- | --- |
| 1 | FT011 | 1 | 1 | 1 | 1 | 1 | 0 | 1 | 1 | 1 | 8 | Lower concern |
| 2 | FT054 | 1 | 1 | 1 | 1 | 0 | 0 | 1 | 1 | 0 | 6 | Moderate/high concern |
| 3 | FT039 | 1 | 1 | 1 | 1 | 1 | 0 | 1 | 1 | 1 | 8 | Lower concern |
| 4 | FT006 | 1 | 0 | 1 | 1 | 0 | 0 | 1 | 1 | 1 | 6 | Moderate/high concern |
| 5 | FT049 | 1 | 1 | 1 | 1 | 0 | 0 | 1 | 1 | 0 | 6 | Moderate/high concern |
| 6 | FT044 | 1 | 0 | 1 | 1 | 0 | 0 | 1 | 1 | 0 | 5 | Moderate/high concern |
| 7 | FT014 | 1 | 1 | 1 | 1 | 1 | 0 | 1 | 1 | 1 | 8 | Lower concern |
| 8 | FT037 | 1 | 1 | 1 | 1 | 1 | 1 | 1 | 0 | 0 | 7 | Lower concern |
| 9 | FT031 | 1 | 1 | 1 | 1 | 1 | 0 | 1 | 1 | 0 | 7 | Lower concern |
| 10 | FT046 | 1 | 0 | 1 | 1 | 0 | 0 | 1 | 1 | 0 | 5 | Moderate/high concern |
| 11 | FT073 | 1 | 1 | 1 | 1 | 0 | 0 | 1 | 1 | 0 | 6 | Moderate/high concern |
| 12 | FT004 | 1 | 0 | 1 | 1 | 0 | 0 | 1 | 1 | 1 | 6 | Moderate/high concern |
| 13 | FT034 | 1 | 0 | 1 | 1 | 0 | 0 | 1 | 1 | 0 | 5 | Moderate/high concern |
| 14 | FT058 | 0 | 1 | 1 | 1 | 0 | 0 | 1 | 0 | 0 | 4 | Moderate/high concern |

Supplementary Table S3B. JBI case-series domain assessment for single-arm/noncomparative cohorts

JBI domains: Q1 = clear inclusion criteria; Q2 = condition/outcome measured in a standard and reliable way; Q3 = valid method for identifying eligible cases; Q4 = consecutive inclusion; Q5 = complete inclusion; Q6 = demographic characteristics reported; Q7 = clinical information reported; Q8 = outcomes/follow-up clearly reported; Q9 = setting/site information reported; Q10 = statistical analysis appropriate. “Unclear” was used when the available article reporting did not explicitly support a “Yes” judgment.

| **No.** | **Study ID** | **Q1** | **Q2** | **Q3** | **Q4** | **Q5** | **Q6** | **Q7** | **Q8** | **Q9** | **Q10** | **Yes count** | **Interpretation** |
| --- | --- | --- | --- | --- | --- | --- | --- | --- | --- | --- | --- | --- | --- |
| 1 | FT002 | Yes | Yes | Unclear | No | No | Yes | Yes | No | Yes | No | 5 | Notable reporting/sampling concerns |
| 2 | FT003 | Yes | Yes | Yes | No | No | Yes | Yes | Unclear | Yes | Yes | 7 | Notable reporting/sampling concerns |
| 3 | FT005 | Unclear | Yes | Yes | No | No | Yes | Yes | Yes | Yes | Yes | 7 | Notable reporting/sampling concerns |
| 4 | FT010 | Yes | Yes | Yes | No | Unclear | Yes | Yes | Yes | Yes | Yes | 8 | Minor reporting/sampling concerns |
| 5 | FT016 | Yes | Yes | Yes | Unclear | Yes | Yes | Yes | Yes | Yes | Yes | 9 | Minor reporting/sampling concerns |
| 6 | FT017 | Yes | Yes | Yes | Yes | Yes | Yes | Yes | Unclear | Yes | Yes | 9 | Minor reporting/sampling concerns |
| 7 | FT018 | Yes | Yes | Yes | Yes | Yes | Yes | Yes | Unclear | Yes | Yes | 9 | Minor reporting/sampling concerns |
| 8 | FT019 | Yes | Yes | Yes | Yes | Yes | Yes | Yes | Yes | Yes | Yes | 10 | All criteria met |
| 9 | FT023 | Yes | Yes | Unclear | No | No | Yes | Yes | No | Yes | No | 5 | Notable reporting/sampling concerns |
| 10 | FT033 | Unclear | Yes | Yes | No | No | Yes | Yes | Unclear | Yes | Yes | 6 | Notable reporting/sampling concerns |
| 11 | FT035 | No | Unclear | Unclear | No | No | Yes | Yes | No | Yes | No | 3 | Notable reporting/sampling concerns |
| 12 | FT043 | Yes | Yes | Yes | No | No | Yes | Yes | Unclear | Yes | Yes | 7 | Notable reporting/sampling concerns |
| 13 | FT047 | Yes | Yes | Yes | Unclear | Yes | Yes | Yes | Yes | Yes | Yes | 9 | Minor reporting/sampling concerns |
| 14 | FT053 | Yes | Yes | Yes | No | No | Yes | Yes | Unclear | Yes | Yes | 7 | Notable reporting/sampling concerns |
| 15 | FT062 | Yes | Yes | Yes | Unclear | Yes | Yes | Yes | Yes | Yes | Yes | 9 | Minor reporting/sampling concerns |
| 16 | FT064 | Yes | Yes | Yes | No | No | Yes | Yes | Unclear | Yes | Yes | 7 | Notable reporting/sampling concerns |
| 17 | FT070 | Yes | Yes | Unclear | No | No | Yes | Yes | No | Yes | No | 5 | Notable reporting/sampling concerns |
| 18 | FT071 | Yes | Yes | Yes | Unclear | Unclear | Yes | Yes | Yes | Yes | Yes | 8 | Minor reporting/sampling concerns |
| 19 | Li X 2024 | Yes | Yes | Yes | No | No | Yes | Yes | Unclear | Yes | Yes | 7 | Notable reporting/sampling concerns |

Supplementary Table S3C. Interpretation note

| Assessment component | Category / concern | Count | Interpretation |
| --- | --- | --- | --- |
| NOS comparative nonrandomized studies | Domain-level NOS assessment | 14 studies | Scores displayed by domain; no official low/moderate/high cutoff imposed. |
| JBI noncomparative cohorts/case series | Domain-level JBI assessment | 19 studies | Yes/No/Unclear/NA displayed by item; Yes count is descriptive. |
| RoB 2 randomized study | Domain-level RoB 2 assessment | 1 study | FT059 judged high overall risk because D1 was high. |

Supplementary Table S4. GRADE certainty assessment for the overlap-adjusted primary outcome

| Outcome | Studies / participants | Pooled estimate | Risk of bias | Inconsistency | Indirectness | Imprecision | Publication bias / small-study effects | Overall certainty |
| --- | --- | --- | --- | --- | --- | --- | --- | --- |
| Actual completed hepatectomy rate | 34 studies; 5,137 patients | 25.40% (95% CI, 20.74%-30.69%); PI 7.16%-60.04% | Serious | Very serious | Serious | Not serious | Not serious; Egger P=0.381 | Very low |

Footnotes: Certainty in the pooled descriptive proportion was appraised using GRADE domains for an observational evidence base. Evidence began at low certainty and was rated down for serious risk of bias, very serious inconsistency (I²=91.5%, tau²=0.5132, prediction interval 7.16%-60.04%), and serious indirectness because definitions of unresectability, conversion intent, and surgical eligibility varied. Imprecision was not rated down separately because the pooled 95% CI was comparatively narrow, while the wide prediction interval was considered under inconsistency. Publication bias/small-study effects were not rated down separately because Egger's regression was not statistically significant (P=0.381), although undetected selective reporting remains possible. Overall certainty was very low.

Supplementary Table S5. Full-text exclusion categories and representative reports

The table summarizes all 38 database reports excluded after full-text review and identifies representative near-eligible reports that could otherwise appear to meet the review criteria.

| **Full-text exclusion category** | **n** | **Reason** | **Representative near-eligible report(s)** | **DOI/source** | **Note** |
| --- | --- | --- | --- | --- | --- |
| Non-original or insufficient report | 10 | Review, abstract, protocol, or insufficient peer-reviewed data | Nagamatsu et al. 2015 | 10.1002/hep.28212 | Conference abstract |
| Endpoint mismatch or no extractable completed hepatectomy | 11 | Radiological conversion or surgical eligibility without a completed-resection numerator for an all-treated denominator | Excluded screening records | Review records | FT020 was not counted here; it remained qualitative-only. |
| Duplicate or overlapping cohort/source | 7 | A more complete canonical report was retained to avoid double counting | Overlapping companion reports (canonical report retained) | Screening/source-adjudication records retained by review team | Canonical reports were retained; overlapping companion reports were excluded to avoid double counting. |
| No relevant conversion-therapy strategy | 3 | Intervention did not meet the conversion/downstaging eligibility definition | Tang et al. 1991 | 10.1007/BF02988567 | Ineligible strategy |
| No all-treated denominator or post-conversion surgery-only cohort | 3 | Denominator was conditioned on successful conversion or surgery | Zhang et al. 2023; Sun et al. 2026 | 10.1186/s12885-023-10955-7; 10.1186/s12876-026-04677-w | Could not estimate baseline resection probability |
| Wrong disease or HCC denominator unconfirmed | 2 | HCC-specific eligible participants could not be separated | Screening records | Retained by review team |  |
| Ineligible or mixed population not separable | 2 | Eligible unresectable HCC data could not be separated | Screening records | Retained by review team |  |
| Total | 38 | Matches the database-channel full-text exclusions in Figure 2 |  |  |  |

Note: Exclusion categories sum to 38 reports and match the PRISMA flow diagram. Detailed screening decisions and source records are retained by the review team.

Supplementary Table S6. Descriptive summary of secondary outcomes across all 36 unique studies and reasons for not pooling

Extraction principle. Two authors independently reviewed eligible reports and extracted synthesis-critical data. Rows 1-34 correspond to the final primary quantitative dataset. Rows 35-36 are the two qualitative-only unique studies. The linked Li X 2023 and Li X 2024 publications describe the same 94-patient cohort and therefore count as one unique study; Li X 2024 is the canonical primary report and Li X 2023 supplies secondary context. Final consensus values are presented below.

| **No.** | **Study** | **Review cohort N / resection status** | **Secondary outcomes** | **Secondary-outcome denominator** | **Reported secondary data** | **Reason not pooled** |
| --- | --- | --- | --- | --- | --- | --- |
| 1 | Lu YC 2024 | 51 / 13 | Response; surgery; short-term survival/follow-up; AEs | All-treated denominator (51) for response and resection | CR 4/51, PR 27/51, SD 14/51, PD 6/51; ORR 60.8% and DCR 88.2%; 13/51 underwent resection; median follow-up 14.2 months; adverse events mainly grade 1–2. | Not pooled because survival time points and response definitions were reported heterogeneously. |
| 2 | Bai Z 2024 | 31 / 7 | Response; pCR; pPR; AEs; follow-up | Treatment n=31; surgery/transplant/pathology-selected subsets | ORR 80.65%. Fourteen became eligible for surgery: 7 underwent resection and 7 transplantation. The article reported pCR 6/31 and pPR 8/31 against the treatment cohort. | Resection and transplantation were combined, and pathology percentages did not use a uniform resection-only denominator. |
| 3 | Zhang X 2026 | 142 / 55 | SLR; ORR; OS; EFS; RFS; pathological response; adverse events | Nonrandom treatment arms: LEN-TAP 71 and TACE 71; pathology/RFS among resected patients | LEN-TAP vs TACE: resection 42/71 vs 13/71; ORR 78.9% vs 16.9%; median EFS 20.03 vs 6.52 months and RFS 36.6 vs 19.0 months; MPR 30 vs 3 and pCR 17 vs 0 among resected patients. | Prospective nonrandomized study; arm-level data were separated in the strategy analysis and pathology was surgery-selected. |
| 4 | Pan X 2023 | 49 / 17 | OS; PFS; ORR; DCR; AEs | Response-evaluable denominator for ORR/DCR (42/49); all-treated denominator for resection | ORR 57.1% by mRECIST and 14.3% by RECIST v1.1; DCR 92.9% and 88.1%; 17/49 underwent resection; median OS and median PFS not reached; grade ≥3 AEs in 36.7%. | ORR denominator differs from all-treated denominator; survival immature/not reached. |
| 5 | He C 2022 | 33 / 19 | OS; resection details | Treatment-arm denominators: PVE+TACE group 33 and TKIs+TACE comparator 64 | PVE+TACE group: 19/33 radical resections; comparator: 0 resections; mean/median OS 736.5/425.0 days vs 424.5/344.0 days; OS favored PVE+TACE (P=0.023). | Comparator treatment-arm survival, not a pooled all-treated secondary outcome. |
| 6 | Su Y 2026 | 210 / 47 | Predictors of surgical conversion; nomogram performance | All-treated cohort 210; training 147 and internal validation 63 | Resection 47/210 (22.4%). Lower tumour burden score, NLR and CAR, and absence of cirrhosis were associated with conversion. AUC was 0.850 in training and 0.871 in internal validation. | Prediction-model outcomes were not treatment-effect or survival outcomes; validation was internal only. |
| 7 | Zhang YQ 2016 | 831 / 43 | OS | Downstaged-resectable subset; surgery vs non-surgery groups | Median OS 49 vs 31 months; 2-, 4-, and 5-year OS 93%, 47%, 26% vs 74%, 18%, 10%; MVI subgroup median OS 58 vs 30 months. | OS reported only in 82 downstaged resectable patients, not all 831 TACE-treated patients. |
| 8 | Lin SR 2025 | 301 / 63 | OS; PFS; pCR; R0; radiological CR; response | All-treated cohort for resection; surgery-selected and radiological-response subgroups for pCR/survival | 301 patients; 63/301 resected; pCR 20/63 (31.7%) among resected patients; R0 resection 100%; surgery group OS not reached vs 58.5 months and PFS 42.83 vs 9.7 months compared with non-surgery group; radiographic CR associated with better OS/PFS. | Survival compares selected surgery vs non-surgery groups; pCR denominator resected only. |
| 9 | Wu XK 2024 | 55 / 29 | ORR; DCR; MPR; pCR; AEs; follow-up | All-treated denominator for ORR/DCR; surgery/pathology denominator for MPR/pCR | 55 patients; median follow-up 13.3 months; CR 9, PR 33, SD 5, PD 7; ORR 76.4% and DCR 85.5% by mRECIST; 29 underwent resection; R0 28/29; MPR 19/29 and pCR 6/29; grade 3–4 TRAEs 43.6%. | Pathological outcomes are surgery-selected; single-arm prospective cohort. |

Qualitative-only status. FT020 was not pooled because completed hepatectomy was not explicitly documented for the reported 3/26 conversion-resection endpoint. Wei et al. 2021 was retained descriptively because independence from a larger multicenter network cohort could not be confirmed. These two studies are not included in the 5,137-patient/1,042-event primary denominator.

| **No.** | **Study** | **Review cohort N / resection status** | **Secondary outcomes** | **Secondary-outcome denominator** | **Reported secondary data** | **Reason not pooled** |
| --- | --- | --- | --- | --- | --- | --- |
| 10 | Zhang W 2023 | 56 / 23 | OS; PFS; response; pCR; MPR | All 56 treated patients; protocol and patient-level surgery reporting were reconciled | The report described 21 protocol-defined successful-conversion surgeries plus two additional completed resections identified in patient narratives (one outside the protocol success classification and one after prolonged treatment). | All 23 documented completed hepatectomies were included in the primary endpoint. |
| 11 | Wang L 2023 | 36 / 12 | EFS; PFS; ORR; DCR; recurrence; AEs | All 36 treated patients; post-treatment outcomes used selected denominators | ORR was 36.1% and DCR was 94.4% by RECIST v1.1. Eleven patients underwent initial radical surgery, one later underwent salvage resection, and one different patient received RFA plus SBRT. | The salvage resection was included as a completed hepatectomy; RFA plus SBRT was not counted as resection. |
| 12 | Regnault H 2024 | 127 / 11 | OS; PFS; downstaging/curative treatment | All 127 SIRT-treated patients for downstaging; selected subgroups for curative treatment/survival | Downstaging occurred in 50/127; 29 received curative treatment (17 transplantation, 11 resection, 1 RFA). Median OS/PFS was 51/47 vs 10/11 months in downstaged vs non-downstaged groups. | Curative treatment combined transplantation, resection and RFA; survival used post-treatment subgroups. |
| 13 | Zhang YC 2023 | 42 / 13 | Tumor response; ORR; DCR; conversion surgery; pathology; postoperative outcomes; OS | Response/OS: all 42 patients; pathology/postoperative outcomes: 13 resected patients (12 followed) | CR/PR/SD/PD 9/21/8/4; ORR 71.4%, DCR 90.5%; 13 radical resections; pCR 7/13 and residual viable tumour <10% in 2/13. Overall 6-, 12-, and 18-month OS was 100%, 91.7%, and 65.0%; postoperative 1-year survival was 83.3% among 12 followed. | Pathology and postoperative outcomes were surgery-selected; residual tumour <10% was not explicitly defined as MPR; recurrence/RFS/DFS was not extractable. |
| 14 | Yang DL 2025 GUIDANCE001 | 802 / 233 | OS; PFS; response; hepatectomy; pathological complete response; serious adverse events | Treatment arms: triple therapy 343 and TACE alone 459; resected-patient denominator for pCR | Triple therapy vs TACE: OS HR 0.43; median PFS 15.9 vs 8.0 months; hepatectomy about 125/343 vs 108/459 (aggregate 233/802); pCR 32.1% vs 11.1% among resected patients. | Treatment arms were separated for strategy analysis; pathology used a resected-patient denominator; the erratum was considered. |
| 15 | Du Y 2023 | 29 / 2 | Tumor response; treatment efficacy; safety | All treated cohort; treatment-arm denominators where reported | The Chinese-language comparative report provided treatment-response and safety information in addition to 2 completed hepatectomies among 29 treated patients. | Outcome definitions and treatment-arm denominators were not sufficiently harmonized for secondary meta-analysis. |
| 16 | Li J 2023 | 87 / 10 | OS; PFS; ORR; DCR; pCR; MPR; AEs | All-treated denominator for response/survival; surgery/pathology denominator for pCR/MPR | 87 patients; median OS not reached; median PFS 10.5 months; ORR/DCR 71.3%/89.7% by mRECIST and 35.6%/87.4% by RECIST 1.1; 10 patients underwent R0 resection; 2 pCR and 4 MPR. | Pathological response only among resected patients; survival is single-arm all-treated cohort. |
| 17 | He ZL 2026 | 45 / 13 | Downstaging conversion; actual resection; safety | All 45 treated patients | All patients received sintilimab, anlotinib and TACE. Thirteen of 45 patients (28.89%) were successfully downstaged to resectable HCC and underwent resection. The publisher abstract describes the treatment as having acceptable safety. | Detailed survival, pathology, recurrence and postoperative-complication data were not included in the accessible official abstract and should not be inferred. |
| 18 | Piao M 2025 | 613 / 74 | OS; PFS; postoperative treatment; recurrence-related outcomes | All 613 treated patients for resection; 128 post-treatment surgery-eligible patients for survival | Of 128 patients meeting resection criteria, 74 underwent surgery and 54 did not. Surgery vs non-surgery median PFS was 29.4 vs 11.2 months (HR 0.39) and median OS was not reached vs 25.4 months (HR 0.26). | Survival compared a post-treatment selected surgery-eligible subgroup, not the full all-treated cohort; it was not interpreted causally. |

| **No.** | **Study** | **Review cohort N / resection status** | **Secondary outcomes** | **Secondary-outcome denominator** | **Reported secondary data** | **Reason not pooled** |
| --- | --- | --- | --- | --- | --- | --- |
| 19 | Li B 2021 | 83 / 24 | OS; PFS; ORR; AEs | Treatment-arm denominators: TACE-HAIC 41 vs c-TACE 42 | Conversion 48.8% vs 9.5%; ORR 14.6% vs 2.4% by RECIST and 65.9% vs 16.7% by mRECIST; median PFS not reached vs 9.2 months; median OS not reached vs 13.5 months. | Treatment-arm outcome comparison; response criteria and survival endpoints not comparable across studies. |
| 20 | Zhang J 2021 | 34 / 14 | ORR; CR/PR; pCR; safety | Response-evaluable denominator (25/34); surgery/pathology-selected denominator | Best ORR 96.0% (24/25): CR 48.0% and PR 48.0%; surgical conversion 15/25; surgical resection 14/25; pCR reported in 7 patients; toxicities manageable. | Only 25/34 completed treatment evaluation; pCR is pathology/surgery-selected. |
| 21 | Yang YG 2023 | 69 / 12 | PFS; OS; ORR; surgery; AEs | Treatment-arm denominators: TAHPLa 37 and SoraTACE 32 | Surgery conversion 30% vs 3%; ORR by mRECIST 70% vs 31%; median PFS 10.3 vs 5.1 months; median OS 27.8 vs 10.7 months; no treatment-related deaths. | Treatment-arm comparison; different regimen and comparator. |
| 22 | Zeng ST 2025 | 84 / 10 | PFS; ORR; DCR; conversion surgery; pCR; adverse reactions | Treatment arms: HAIC+lenvatinib+PD-1 inhibitor 48 and HAIC+lenvatinib 36; pathology among resected patients | Triple vs double therapy: median PFS 10.6 vs 5.2 months; ORR 70.8% vs 66.7%; DCR 93.8% vs 86.1%. Five patients per arm underwent surgery; pCR was 4/5 vs 1/5. | Comparative treatment-arm outcomes and surgery-selected pathology denominators were not directly poolable. |
| 23 | Yalikun K 2025 | 19 / 9 | ORR; DCR; MPR; pCR; PFS/OS follow-up; treatment-related adverse events | All 19 treated patients for response/resection; 9 resected patients for pathology | Fourteen became resectable; 9 underwent R0 resection. RECIST ORR/DCR 47.4%/89.5%; among resected patients MPR 3/9 and pCR 2/9. Follow-up was 14.9 months; grade >=3 treatment-related events occurred in 14/19. | Small single-arm cohort; pathology was surgery-selected and survival follow-up was immature. |
| 24 | Qu WF 2022 | 51 / 19 | OS; EFS; DFS; ORR; DCR; AEs | Treatment-arm denominators: triple CT 30 and dual CT 21; surgery/upfront cohorts for DFS | ORR 76.7% vs 47.6%; DCR 90.0% vs 57.1%; median OS and EFS not reached in triple-therapy cohort; conversion resection 50.0% vs 19.0%; DFS after conversion resection compared with upfront-surgery cohort. | Mixes conversion cohort and upfront-surgery comparator; DFS uses surgery-selected denominator. |
| 25 | Son SY 2024 | 26 / 1 | Downstaging to curative-intent treatment; response; follow-up | All 26 Y-90-treated patients; curative-treatment subgroups where reported | The cohort evaluated conversion to transplant or surgical resection after boosted-dose yttrium-90 radiation segmentectomy; 1 completed liver resection was used for the primary endpoint. | Curative treatment combined transplantation and resection, and the very small cohort precluded a harmonized secondary synthesis. |
| 26 | Chen S 2022 | 142 / 26 | OS; PFS; ORR; DCR; AEs | Treatment-arm denominators in 142 eligible PD-L1-positive uHCC patients | Follow-up 27 months; conversion 25.7% vs 11.1%; median OS 18.1 vs 14.1 months; median PFS 9.2 vs 5.5 months; ORR 47.1% vs 27.8%; DCR 70.0% vs 52.8%. | Treatment-arm comparison; denominator and treatment selection differ from pooled resection-rate endpoint. |
| 27 | Zeng ZC 1998 | 65 / 20 | Tumor response; surgery after radioimmunotherapy; long-term survival | All treated cohort and surgery-selected follow-up | The historical phase I/II report described survival and subsequent surgery after intrahepatic arterial 131I-anti-HCC monoclonal-antibody therapy; 20/65 completed resections were used for the primary endpoint. | Historical treatment context and surgery-selected survival reporting were not comparable with contemporary secondary endpoints. |

| **No.** | **Study** | **Review cohort N / resection status** | **Secondary outcomes** | **Secondary-outcome denominator** | **Reported secondary data** | **Reason not pooled** |
| --- | --- | --- | --- | --- | --- | --- |
| 28 | Chen MS 2002 | 473 / 72 | Tumor response; survival; resection after TACE; treatment-related outcomes | Randomized treatment arms; all-treated denominator for resection | The randomized historical TACE study reported completed liver resections together with response and survival outcomes across treatment arms; 72/473 completed resections were used for the primary endpoint. | Arm-specific historical regimens, time origins, and secondary-outcome definitions were not harmonizable with later studies. |
| 29 | Liu D 2023 | 29 / 14 | Response; surgery; pathology; safety | Enrolled/evaluable n=29; surgical/pathology reporting used a 19-patient subset | ORR 17/29 (58.6%). Nineteen patients became eligible for further treatment and 14 completed resection; pCR 1/19 and major partial response 5/19 were reported using 19 as the pathology denominator. | Enrollment, treatment-eligibility, resection, and pathology denominators were not aligned. |
| 30 | Gan LJ 2023 | 98 / 13 | OS; PFS; ORR; DCR; pCR; AEs | Potentially resectable subgroup (PRP, n=37) and non-potentially resectable subgroup (NPRP, n=61) | PRP ORR was 67.6% by RECIST and 75.7% by mRECIST. Fifteen successful local treatments comprised 13 hepatectomies and 2 RFA procedures; pCR occurred in 3 resected patients. Median follow-up was 28 months; PRP versus NPRP median PFS was 25 versus 13 months and median OS was not reached versus 21 months. | The conversion-treatment count included two RFA procedures; only the 13 completed hepatectomies were counted as primary events. |
| 31 | Cao YJ 2023 | 20 / 14 | OS; EFS; RFS; ORR; DCR; MPR; pPR; AEs | All-treated denominator for ORR/DCR/survival; surgery/pathology denominator for MPR/pPR | 20 patients; conversion success 70.0%; MPR 7 and pPR 6 among resected/pathology-evaluable patients; ORR 75.0%; DCR 95.0%; median EFS 9.46 months; median OS 19.48 months; median RFS 9.63 months; grade 3 TRAEs 25.0%. | Pathological endpoints use resected/pathology-evaluable denominator; single-arm small cohort. |
| 32 | Xu B 2022 | 187 / 29 | Potential resectability criteria; treatment response; conversion surgery; predictors | All treated cohort for resection; predictor-defined subgroups | The report evaluated criteria associated with potential resectability during lenvatinib plus anti-PD-1 therapy and documented 29 completed resections among 187 treated patients. | Predictor-defined subgroup analyses and resectability criteria were not pooled as treatment effects or survival outcomes. |
| 33 | Zhou Y 2025 | 123 / 49 | Early tumour shrinkage; conversion resection; OS; treatment-related adverse events | Treatment arms: TT 103 and TTR 20 | TTR vs TT: early tumour shrinkage 85.0% vs 59.2%; conversion resection 65.0% vs 35.0% (approximately 13/20 vs 36/103; aggregate 49/123); median OS not reached vs 31.9 months. Treatment-related adverse events were described as manageable. | Small radiotherapy arm and retrospective treatment allocation; both arms nevertheless combine locoregional and systemic therapy. |
| 34 | Li X 2024 (canonical; companion Li X 2023) | 94 / 32 | OS; PFS; early response; conversion surgery | All-treated cohort; early-responder subgroup; surgery-selected subgroup | Early response in 68/94 (72.3%); conversion surgery rate higher in early responders (44.1% vs 7.7%); early responders had longer PFS 15.4 vs 7.8 months and OS 23.1 vs 12.5 months; early responders with surgery had OS/PFS not reached versus non-surgery subgroup. | Linked reports describe the same 94-patient cohort; counted once in the review. Secondary outcomes are retained descriptively, while survival comparisons are observational and surgery-selected. |
| 35 | FT020 (qualitative only) | 26 / not pooled | Reported conversion-resection/surgical-treatment criteria | All 26 treated patients | The source reported a 3/26 conversion-resection endpoint defined through attainment of surgical-treatment criteria, but the available full text did not explicitly document that these patients underwent completed hepatectomy. | Primary endpoint mismatch: completed hepatectomy could not be confirmed; retained qualitatively and only in a broader endpoint sensitivity analysis. |
| 36 | Wei et al. 2021 (qualitative only) | 22 / 10 (descriptive only) | PFS; OS; ORR; DCR; pCR; recurrence | All-treated denominator for ORR/DCR; resected-patient denominator for pCR and postoperative recurrence | ORR 81.8% (18/22); DCR 90.9% (20/22); median PFS and OS not reached; pCR 3 among 10 converted/resected patients; postoperative median follow-up 3.5 months with no recurrence or death. | Potential cohort overlap with a larger multicenter network cohort could not be excluded; retained descriptively and excluded from primary pooling to avoid possible double counting. |

**Supplementary Table S7. Post hoc treatment-era meta-regression and crude-versus-modeled rate summary**

Because the included publications spanned 1998-2026, publication year was examined as a continuous study-level moderator in a post hoc exploratory random-effects meta-regression. This analysis complements the historical-study sensitivity analyses and should be interpreted only as an ecological proxy for treatment era.

| **Analysis / metric** | **Estimate** | **95% CI / interval** | **Heterogeneity / test** | **Interpretation** |
| --- | --- | --- | --- | --- |
| Crude aggregate completed-hepatectomy rate | 20.3% (1,042/5,137) | Not model-based | — | Direct aggregation of all events and all-treated denominators. |
| Primary random-effects modeled rate | 25.40% | 20.74%-30.69% | I²=91.5%; tau²=0.5132 | Average study-level probability under a DL random-effects logit model; not identical to the crude aggregate rate. |
| Publication-year meta-regression slope | 0.0170 log-odds/year | -0.0262 to 0.0602 | Qm=0.60; P=0.440; residual tau²=0.5076 | No clear linear study-level association detected; limited power and ecological interpretation. |
| Publication-year effect per decade | OR 1.19 | 0.77-1.83 | Same meta-regression | Publication year cannot isolate changes in regimen, imaging, patient selection, surgery, or reassessment practices. |
